# *GsMYB7* encoding a R2R3-type MYB transcription factor enhances the tolerance to aluminum stress in soybean (*Glycine max* L.)

**DOI:** 10.1186/s12864-022-08744-w

**Published:** 2022-07-22

**Authors:** Hongjie Wang, Xiangli Yin, Dan Du, Zhongyi Liang, Zhenzhen Han, Hai Nian, Qibin Ma

**Affiliations:** 1 The State Key Laboratory for Conservation and Utilization of Subtropical Agro-Bioresources, South China Agricultural University, Guangzhou, China; 2 The Key Laboratory of Plant Molecular Breeding of Guangdong Province, College of Agriculture, South China Agricultural University, Guangzhou, China; 3 The Guangdong Subcenter of the National Center for Soybean Improvement, College of Agriculture, South China Agricultural University, Guangzhou, China; 4 The Guangdong Provincial Laboratory of Lingnan Modern Agricultural Science and Technology, South China Agricultural University, Guangzhou, China; 5 Zengcheng Teaching and Research Bases, South China Agricultural University, Guangzhou, Guangdong 510642 People’s Republic of China

**Keywords:** *GsMYB7*, R2R3-MYB, Transcription factor, Soybean, Acidic aluminum

## Abstract

**Background:**

MYB transcription factor (TF) is one of the largest families of TFs in plants and play essential roles in plant growth and development, and is involved in responses to biological and abiotic stress. However, there are few reports on *GsMYB7* gene in soybean under aluminum acid stress, and its regulatory mechanism remains unclear.

**Results:**

The GsMYB7 protein is localized in the nucleus and has transcriptional activation ability. Quantitative real-time PCR (qRT-PCR) results showed that *GsMYB7* held a constitutive expression pattern rich in roots. When AlCl_3_ concentration was 25 µM, the total root surface area (SA) of *GsMYB7* transgenic lines were 34.97% higher than that of wild-type Huachun 6 (HC6). While the accumulation of Al^3+^ in root tip of transgenic plants after aluminum treatment was 17.39% lower than that of wild-type. RNA-sequencing analysis indicated that over 1181 genes were regulated by *GsMYB7* and aluminum stress. Among all the regulated genes, the expression levels of glutathione peroxidase, protein kinase, cytochrome and other genes in the transgenic lines were significantly higher than those in wild type by acidic aluminum stress. The bioinformatics and qRT-PCR results showed that 9 candidate genes were induced under the treatments of acidic aluminum stress which were indirectly and/or directly regulated by *GsMYB7*. After AlCl_3_ treatments, the transcripts of these genes in *GsMYB7* transgenic seedlings were significantly higher than those of wide-type HC6.

**Conclusions:**

The results suggested that *GsMYB7* may enhance soybean tolerance to acidic aluminum stress by regulating the downstream genes.

**Supplementary Information:**

The online version contains supplementary material available at 10.1186/s12864-022-08744-w.

## Background

Aluminum (Al) is the third richest element in the earth's crust next to oxygen and silicon [1]. Aluminum affects yield by destroying cells in plants which limits root growth and water uptake [2]. Aluminum toxicity is one of the major abiotic stress factors in the acidic soils, meanwhile there are about 30–40% of acidic soils being used to plant crops which strongly limits to crop production [3]. After aluminum stress, the root system is the first to suffer damage, and the root tip is the main damage location [4]. The lateral root is thick, small and easy to break, which inhibits cell elongation and cell division of the primary root, leading to enlargement and browning of the root tip [5].

MYB TF is a large family of TFs in plants which is widely involved in the growth and development of plant tissues, and responds to abiotic stress processes such as drought, salinization and chilling injury [6]. In addition, MYB TF is also closely related to the quality of some cash crops [7]. The MYB TFs with different functions in plants have a common feature in structure which has a certain similarity and unity in its amino acid sequence and conformation [8]. According to the number of DNA binding domains, the MYB family can be divided into four categories: 1R-MYB, R2R3-MYB, 3R-MYB, and 4R-MYB [9, 10]. AtMYB77 is a key protein in ABA and auxin signaling pathways during lateral root development under drought stress, and can resist drought stress by regulating lateral root growth [11]. Overexpression of *GmMYB92*, *GmMYB76* and *GmMYB177* in *Arabidopsis* can enhance the tolerance of transgenic plants to salt and cold injury [12]. *AtSNRK2.4* cooperating with the MYB family gene *AtMYB21* mediates the response to salt stress by upregulating the expression of downstream stress genes [13]. In tobacco, application of active salicylic acid can enhance the disease resistance by inducing the expression of *NtMYB1* gene and regulating the expression of proteins related to disease resistance [14]. The MYB TF plays an important role not only in stress resistance but also in controlling the formation of plant cell morphology. CPC and Wer, two MYB TFs, play an important role in determining the fate of root epidermal cells by interacting with the same R-type BHLH protein [15]. The *PhMYB1* of morning glories and the *MIXTA* of snapfish are homologous genes which can promote the directed production of specified substances in the cell wall of epidermal cells and regulate the formation of conical cells of petal epidermal cells [16].

In plants, most MYB proteins belong to the R2R3-MYB subfamily consisting of two repeats. R2R3-MYB TFs play important regulatory roles in specific plant growth processes, including primary and secondary metabolism, cell development, and response to abiotic and biological stresses [17]. When *GmMYB181* was overexpressed in *Arabidopsis*, the flower organ morphology, fruit size and plant structure of the transgenic lines were changed with the outward curling of sepals, the decrease of pod size, the increase of lateral branches and the decrease of plant height. These results indicate that R2R3-MYB family members are involved in the development of reproductive organs and played an important role in the regulation of plant architecture [18]. An R2R3-MYB TF, *MdMYB23*, was identified and isolated from apples by transcriptome analysis, which was significantly induced and up-regulated under low temperature stress. *MdMYB23* overexpression in apple callus and *Arabidopsis* enhanced the tolerance of plants to cold stress [19]. In addition, the *PsnMYB108* gene isolated from poplar trees was significantly up-regulated in roots and leaves under salt stress. *PsnMYB108* overexpression could significantly improve the tolerance of tobacco plants to salt stress by increasing the scavenging capacity of reactive oxygen species and proline accumulation [20]. Recent studies indicated that the *GmMYB39* gene containing R2R3 repeats at the N-terminal was involved in the inhibition of soybean isoflavone biosynthesis regulation [21]. Further investigation has found that AP2/ERF and R2R3-MYB TFs were involved in plant response to temperature stress and auxin lipid metabolisms [22].

Soybean is an important food crop which is widely grown in the world. Wild soybeans have many excellent genes that can be crossed with cultivated soybeans to produce new genes and alleles to improve the yield and quality of cultivated soybeans [23]. Wild soybean demonstrates potential resistance to abiotic stresses such as drought, salt stress [24]. Xian cloned *GsAAE3* from wild soybean (*Glycine soja*, BW69 line) and confirmed that *GsAAE3* can indirectly improve the tolerance of wild soybean to Cd and Al by reducing oxalic acid accumulation induced by Cd and Al stress [25]. In addition, *GsMATE* enhances the resistance of transgenic plants to Al toxicity by reducing Al accumulation in *Arabidopsis* roots [26]. Previous studies have shown that MYB TFs play important roles in plant abiotic stress, but there are few reports on the tolerance of MYB TFs to acidic aluminum stress [27]. In this study, the *GsMYB7* gene was cloned from *Glycine soja* BW69 line according to the bio-information from the acidic aluminum gene expression profile. The soybean cultivar HC6 was used as the receptor for genetic transformation. We measured the total root surface area and total Al accumulations of transgenic plants and wild type. The transcriptome analysis was performed using *GsMYB7* transgenic plants under aluminum stress, and downstream genes mediated by *GsMYB7* were identified. Our results further reveal the response of *GsMYB7* gene to acid aluminum stress and its regulatory molecular mechanism.

## Result

### Isolation and bioinformatics analysis of *GsMYB7*

**Fig. 1:**
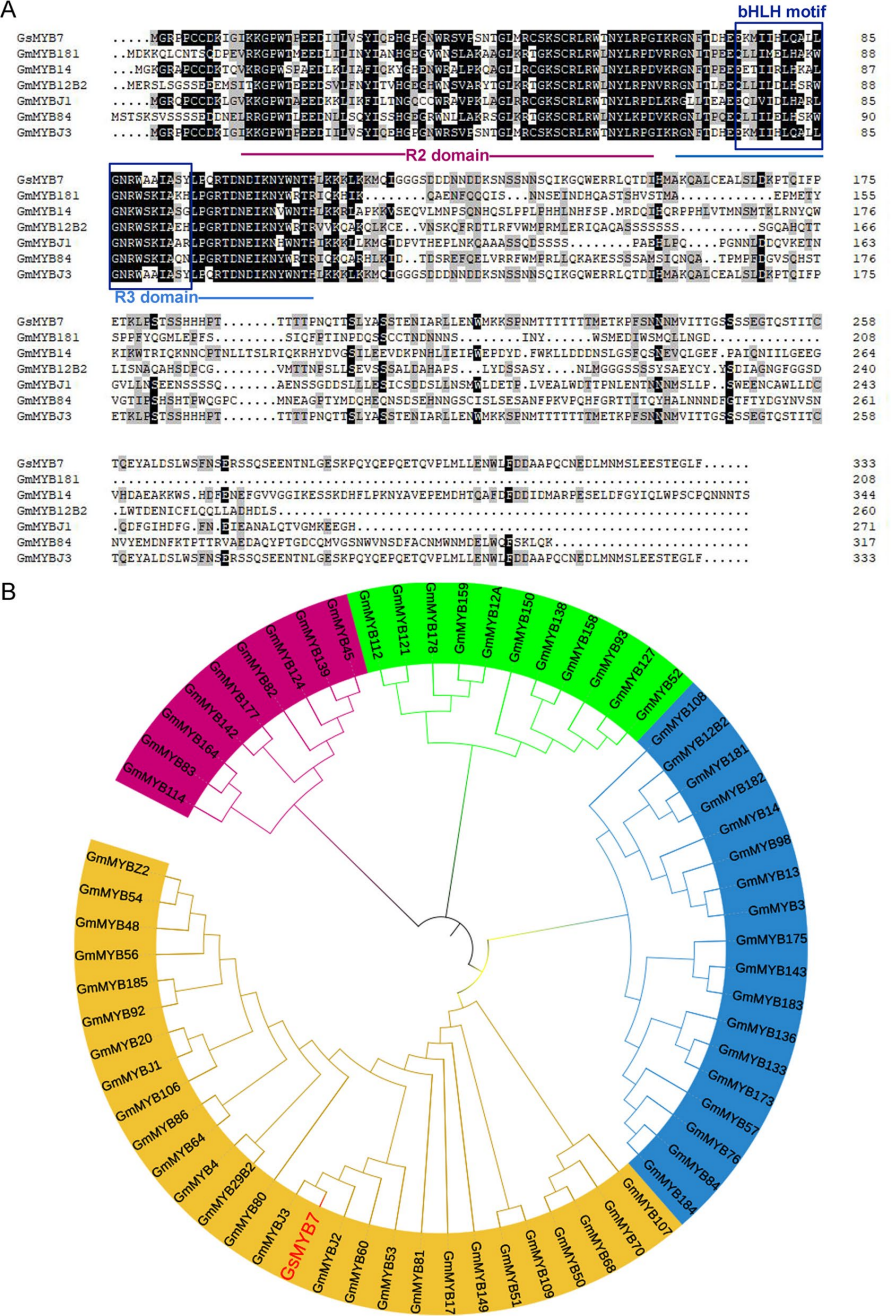
Homology analysis of *GsMYB7* and other MYB transcription factors. **A** Multiple sequence alignment of *GsMYB7* and R2R3 MYB family members from soybean. **B** Phylogenetic tree analysis of GsMYB7 and MYB proteins. The comparison of amino acid sequences was conducted by the software of DNAMAN9.0. The phylogenetic tree was constructed by the neighbor-joining method using the software of MEGA 7.0. All the amino acid sequences of MYB proteins were from NCBI database (https://www.ncbi.nlm.nih.gov/). The detailed information of MYB proteins was available from the Additional file 1: Table S4

The *GsMYB7* gene located on chromosome 6 was cloned from wild soybean BW69 line using specific primers (Additional file 1: Table S3). The length of *GsMYB7* gene was 2179 bp, and the length of open reading frame was 1002 bp encoding 333 amino acids. The sequence alignment revealed that GsMYB7 protein had two conserved DNA binding domains consisting of 46 amino acids and 44 amino acids, respectively. The amino acids were located between 10–60 and 70–120 amino acid residues which was speculated to be the typical structure of R2R3-MYB TF. Phylogenetic tree analysis indicated that the related homologues of *GsMYB7* were mainly distributed in legumes. Though the homology between GsMYB7 protein and its homolog in *Arabidopsis* is not significantly high, both of these genes had MYB DNA binding domains. Through NCBI and Phytozome database, MYB TF family genes were found in soybean. Amino acid sequence alignment and phylogenetic tree analysis indicated that R2R3-MYB TF plays a certain regulatory role in abiotic stress (Fig. 1). For example, soybean lines overexpressing the *GmMYB68* gene increased resistance to salt-alkali stress with higher photosynthetic rates than that of *GmMYB68*-RNAi lines and wild-type. Under normal conditions, there was no significant difference in agronomic traits between wild-type and transgenic plants. However, overexpression of *GmMYB68* significantly increased the number of grains and 100-grain weight of soybean after salt stress [28]. Previous studies had shown that *GmMYBJ1* expression was induced by abiotic stresses such as drought, cold, salt and exogenous abscisic acid (ABA). Compared with wild-type, the *GmMYBJ1* transgenic lines enhanced *Arabidopsis* tolerance to drought and cold stress [29]. In addition, the PLANTCARE database was explored the promoter region of *GsMYB7* gene to discover a few possible cis-elements. The promoter of *GsMYB7* gene with 1500 bp nucleotide sequence upstream of the start codon contains a variety of cis-acting elements including those associated with light, ethylene, auxin, such as AE-box (AGAAACTT), ERE (ATTTCAAA), TGA (AACGAC), GA (ATAGATAA), TCT (TCTTAC), and the like (Additional file 1: Table S2). Therefore, we speculated that GsMYB7 may play potential roles in abiotic stress regulation.

### Subcellular localization and transcriptional activation ability of GsMYB7 protein

**Fig. 2:**
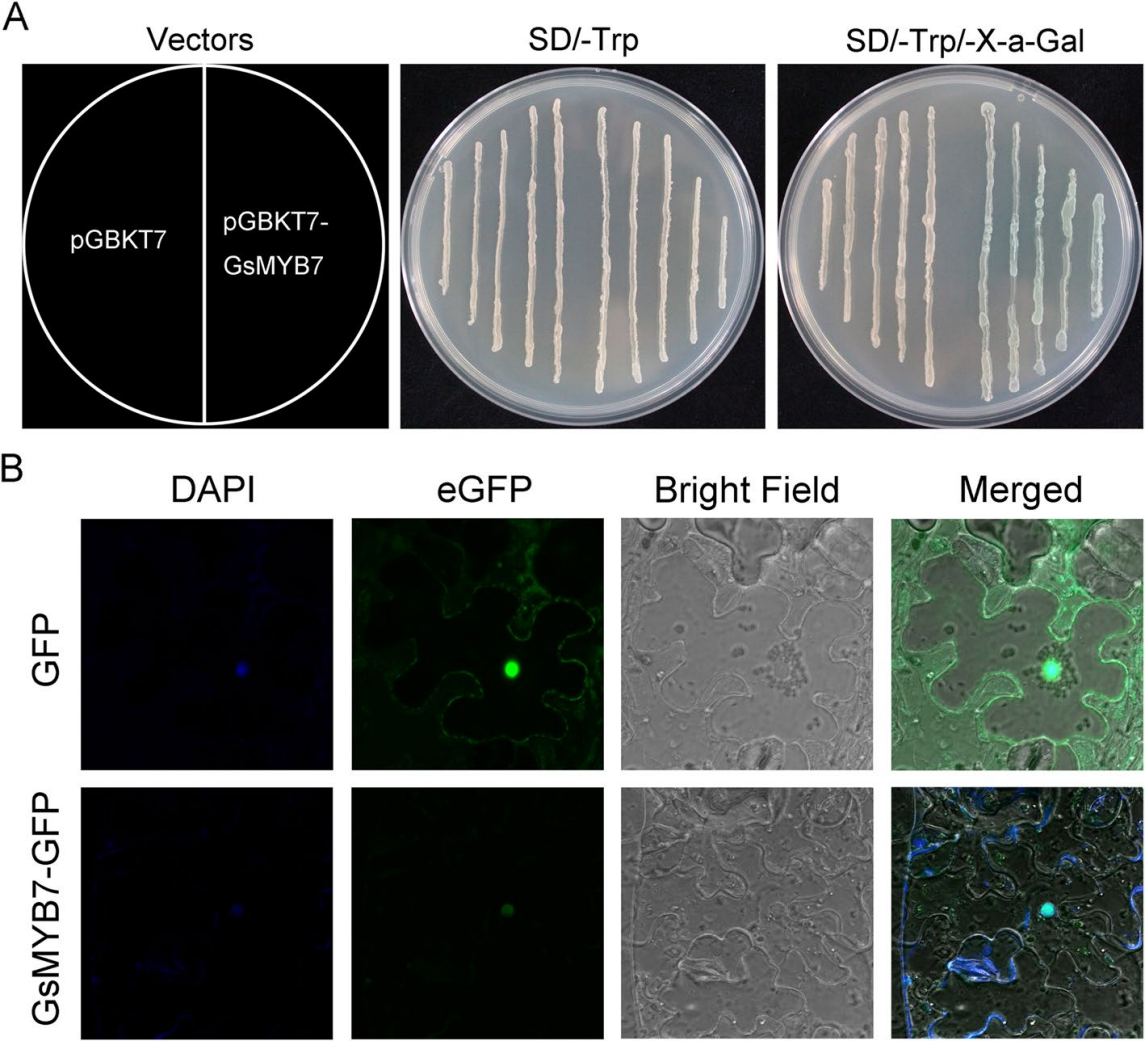
Transcriptional activity detection and subcellular localization of GsMYB7 protein. **A** Transcriptional activation of GsMYB7 protein. **B** Subcellular localization of GsMYB7 protein. The ORF of *GsMYB7* gene was fused with the GAL4 DNA binding region of pGBKT7 vector and expressed in yeast strain Y_2_H Gold. After the transformation, the two positive clones were transferred to SD/-TRP solid medium, and the other group was added with substrate X-a-Gal. The constructed fusion expression vector of pCAMBIA1302-eGFP-GsMYB7 and empty vector pCAMBIA1302-eGFP were transformed into *Agrobacterium tumefaciens* GV3101, respectively, cultured with the virus protein P19 in equal volume, and then injected into 4-week old *Nicotiana tabacum* L epidermal cells. After cultured for 48 h later, the leaves were observed by laser confocal microscopy (Carl Zeiss, Jena, Germany)

The leaf cells of tobacco were used to detect the localization of GsMYB7 protein. The result of laser confocal microscopy showed that GFP protein was ubiquitous in the whole cell, while GsMYB7-GFP fusion protein only had excited fluorescence in the nucleus which overlapped with the chromophore position of the nuclear dye DAPI (Fig. 2B). The results indicated that GsMYB7 is a nuclear localization protein.

Transcriptional activity of GsMYB7 protein showed that positive transformants could grow on the SD/-Trp medium, and the cell accumulation with pGBKT7-GsMYB7 plasmid showed blue color on the solid medium of SD/-Trp-X-α-gal, while that with empty vector showed no color change (Fig. 2A). The results revealed that GsMYB7 protein has the characteristic transcriptional activation.

### *GsMYB7* response to acidic aluminum stress

**Fig. 3:**
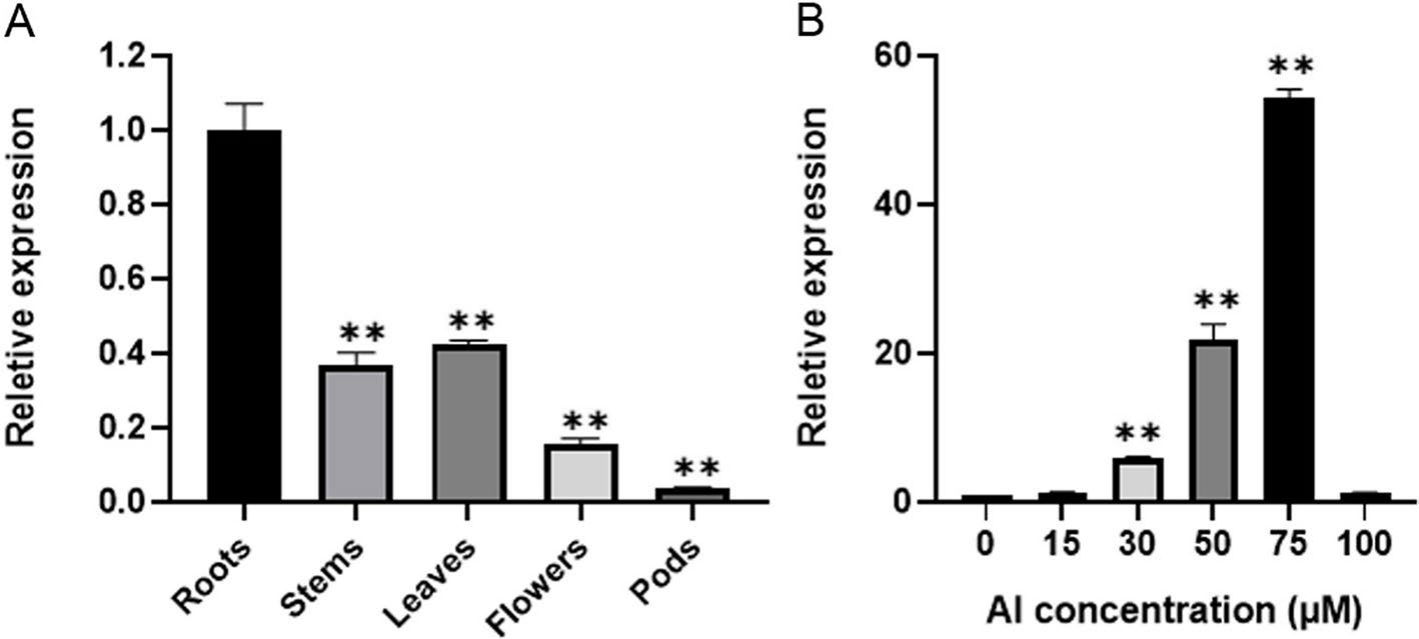
Expression pattern analysis of *GsMYB7*. **A** Tissue expression pattern of *GsMYB7*. The samples were taken from the soybean roots, stems, leaves, flowers and pods of wild soybean BW69 growing under normal nutrient solution. **B** Gene expression of *GsMYB7* in the root of wild soybean BW69. The wild soybean BW69 were cultured for 6 h in the nutrient solutions containing 0, 15, 30, 50, 75 and 100 μM AlCl_3_, respectively. Using soybean *Actin3* as internal reference gene, three independent replicates were detected by 2^^−△△CT^ method. The asterisks in figure A indicate significant differences between roots and other soybean tissues (*P < 0.05; **P < 0.01). The asterisk in figure B indicates that there is a significant difference between 0 μM AlCl_3_ and other Al concentration (15, 30, 50, 75 and 100 μM AlCl_3_) treatment (*P < 0.05; **P < 0.01)

The expression level of *GsMYB7* was detected by taking the roots, stems, leaves, flowers and pods of wild soybean BW69 line as samples. The results of qRT-PCR showed that *GsMYB7* was expressed in all organs of the plant with the highest expression level in roots and the lowest in pods (Fig. 3A). In addition, the expression of *GsMYB7* was up-regulated by acidic aluminum with the concentration gradients of different AlCl_3_ (pH4.5). The transcripts of *GsMYB7* were increased firstly and then decreased with the increase of Al concentrations. Among them, the *GsMYB7* transcripts in 75 μM AlCl_3_ treatment were 54 times those of the control treatment, while the expression of *GsMYB7* was inhibited at high concentration of AlCl_3_ (Fig. 3B).

### Identification of soybean transgenic plants

**Fig. 4:**
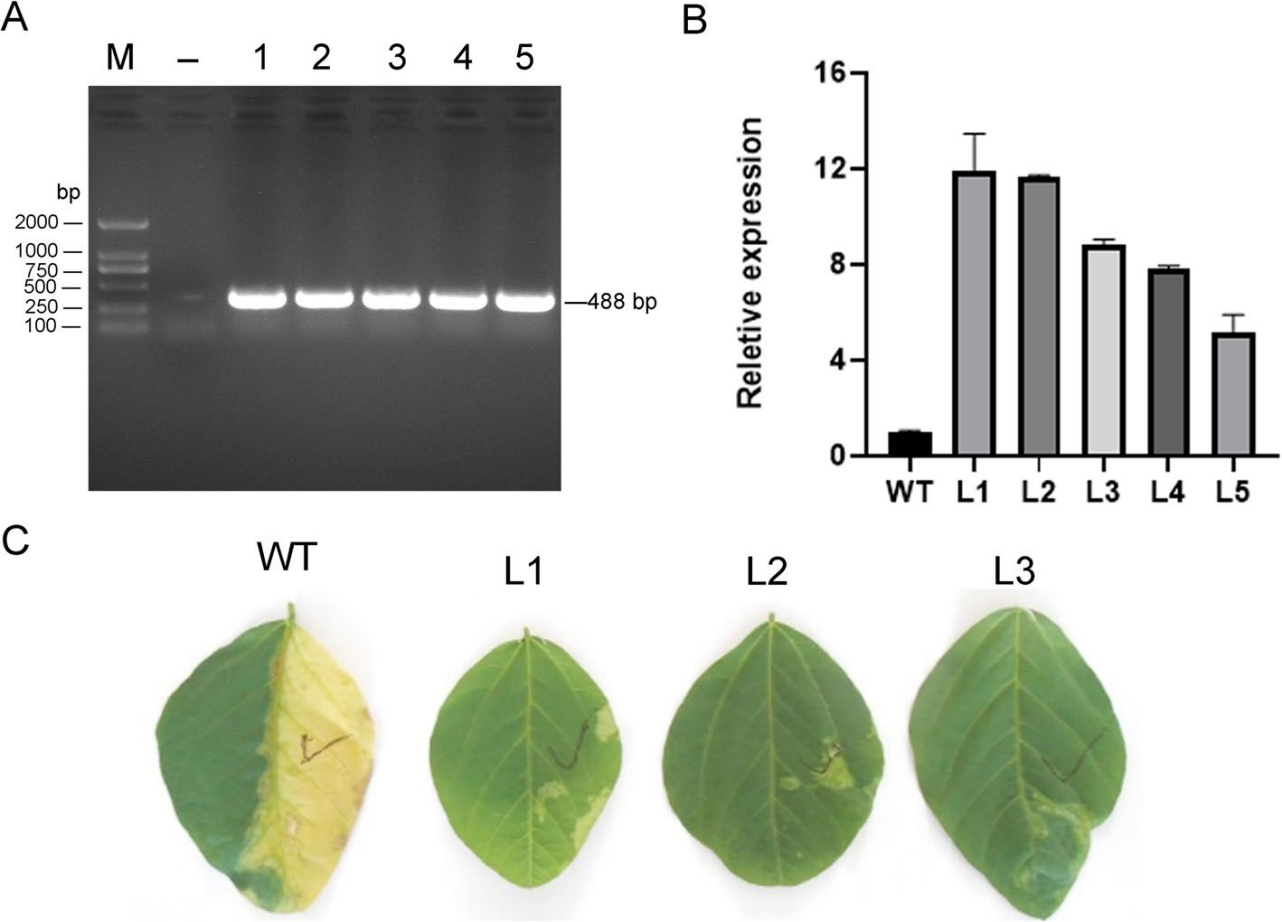
Detection of transgenic soybean lines. **A** PCR identification using *Bar* gene primers (Additional file 3: Table S10). **B** The gene expression of transgenic soybean lines was detected by qRT-PCR. (**C**) Tolerance of transgenic plants to herbicides. The blade side with the tick is the side to which the herbicide is applied, and the left side is untreated with herbicide. The relative gene expression levels of HC6 (WT) and *GsMYB7* transgenic soybean lines were calculated by 2^^−△△CT^ method in three separate biological experiments. WT: wild type, HC6; L1, L2, L3, L4, L5: *GsMYB7* transgenic T_3_ generation soybean

In order to determine whether the *GsMYB7* gene was transferred into soybean HC6, the triple compound leaves of HC6 and the transgenic plants were coated with herbicide Liberty® (Glufosinate ammonium) at a concentration of 135 mg/L for 3 days. The observation indicated that the leaves of soybean HC6 changed from green to yellow,, while the leaves of *GsMYB7* transgenic plants did not change significantly because of herbicide resistance (Fig. 4C). In addition, the *GsMYB7* lines of T_3_ generation were identified by PCR using *Bar* gene primers. The results showed that the *Bar* gene specific band of about 488 bp was detected in the *GsMYB7* overexpressing transgenic lines (Fig. 4A). Then, the expression levels of *GsMYB7* transgenic lines were determined by qRT-PCR. The results showed that *GsMYB7* transgenic lines were rich in the highest levels which were selected for subsequent phenotype identification (Fig. 4B).

### Overexpression of *GsMYB7* enhances aluminum resistance in transgenic soybean

**Fig. 5:**
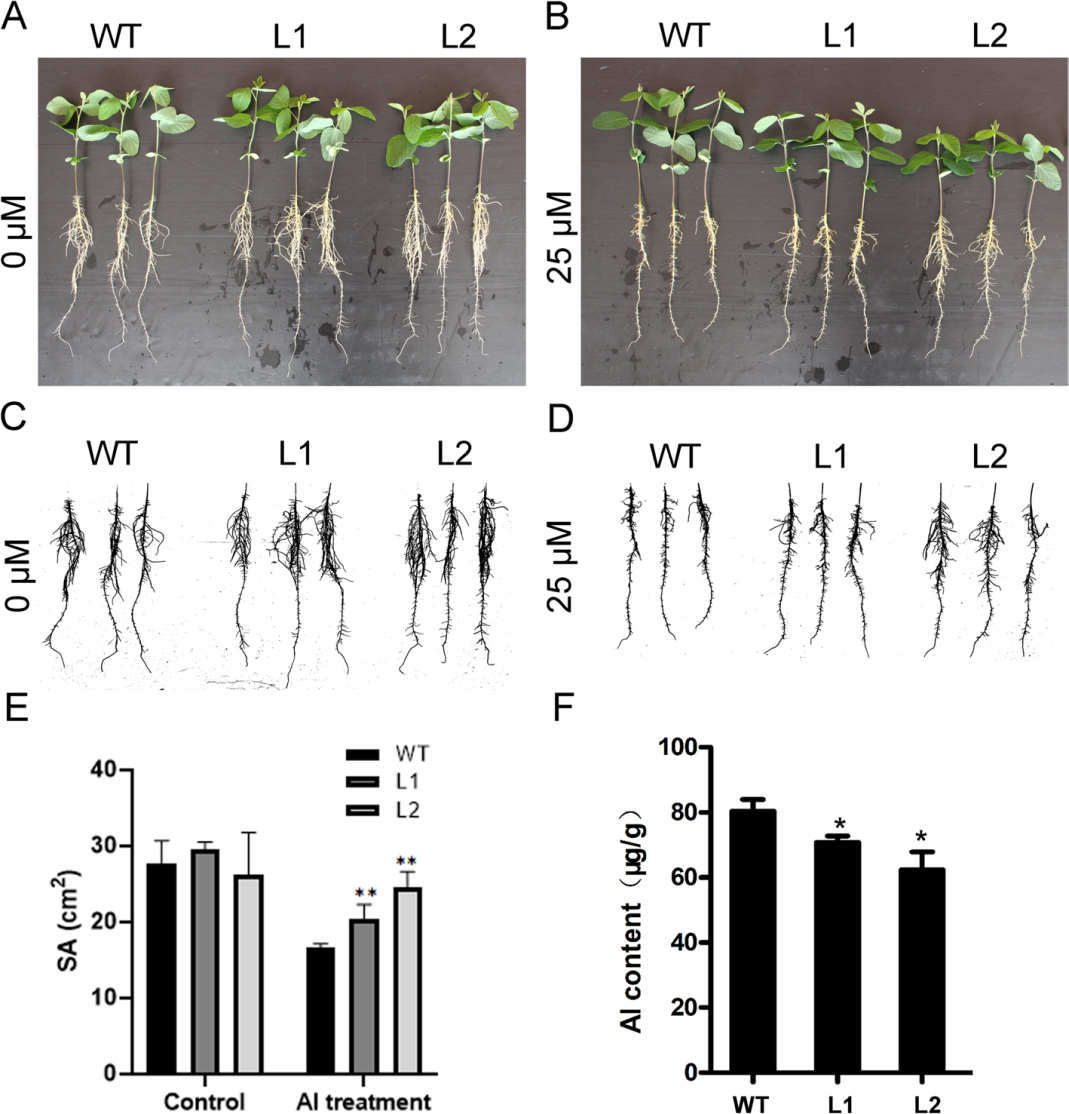
Tolerant phenotype to Al stress of GsMYB7 transgenic lines. **A**, **B** The phenotypes were observed after 5 days of treatment with CaCl_2_ solution containing different concentrations of AlCl_3_. The seedlings were treated with 0, 25 μM AlCl_3_, respectively. **C**, **D** The scanned roots were observed after AlCl_3_ treatment with concentrations of 0 and 25 μM, respectively. **E** The total root area of L1, L2 and WT under 0 and 25 μM AlCl_3_ treatments for 5 days. **F** The total Al accumulations in L1, L2 and WT were determined via ICP-AES analysis. The asterisk indicated that there was significant difference between HC6 and *GsMY*B7 transgenic lines (*P < 0.05; **P < 0.01). WT: wild type, Huachun 6; L1, L2: *GsMYB7* transgenic soybean of T_3_ generation

To study the effects of *GsMYB7* on soybean plants under acidic aluminum stress, the treatments of AlCl_3_ concentrations were designed to observe the root difference between the *GsMYB7* transgenic lines and wild type. The statistical results showed that the total root surface area of the *GsMYB7* transgenic lines and wild type were generally inhibited under the treatments of AlCl_3._ When AlCl_3_ concentration was 25 µM, the total root surface area of *GsMYB7* transgenic lines were 34.97% higher than that of wild-type. Under the same concentration of AlCl_3_, the total root surface area of *GsMYB7* transgenic plants was significantly higher than that of wild type (Fig. 5A-E). Finally, the contents of Al^3+^ in the extracts of soybean roots were determined by inductively coupled plasma atomic emission spectrometry. As shown in Fig. 5F, the aluminum ion contents of the two transgenic lines were less than that of HC6, the content of Al^3+^ in root of transgenic lines was 17.39% lower than that of HC6 (Fig. 5F). The results indicated that the *GsMYB7* transgenic lines were more resistant to acidic aluminum stress than that of HC6.

### Differentially expressed gene analysis

**Fig. 6:**
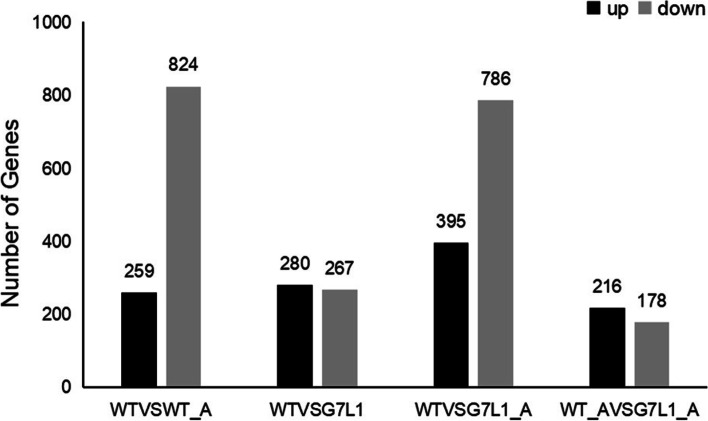
Differential gene statistics. The up-regulated and down-regulated significantly expressed genes in each group of differential expression analysis were statistically analyzed and shown in a bar chart. The black column represents the up-regulated gene frequency and the gray column represents the down-regulated gene frequency. WTVSWT_A: Comparative analysis of differential genes between wild-type (HC6) and wild-type aluminum treatment; WTVSG7L1: Comparative analysis of differential genes between wild-type (HC6) and *GsMYB7* transgenic lines; G7L1VSVSG7L1_a: Comparative analysis of differential genes between *GsMYB7* transgenic lines and GSMYB7 transgenic lines under acidic aluminum treatment; WT_AVSG7L1_A: Comparative analysis of differential genes between wild type (HC6) aluminum treatment and *GsMYB7* transgenic line acidic aluminum treatment

The molecular basis of the difference of aluminum sensitivity between transgenic soybean and wild-type (WT) was studied by RNA-Seq analysis. In order to dig deeper into the RNA sequencing results, we mainly analyzed the RNA sequencing results of WTVSG7L1 group and WT_AVSG7L1_A group (Additional file 2: Table S7, S8). In the WTVSG7L1 group, the expression levels of 547 genes were changed by overexpression of *GsMYB7* gene, among which 267 genes were significantly up-regulated (log2fc > 1). In the WT_AVSG7L1_A group, 394 genes with differential expression levels were obtained from the soybean seedlings of *GsMYB7* transgenic lines and wild type which were treated under the acidic aluminum, among which 178 genes were significantly up-regulated (log2Fc > 1) (Fig. 6).

**Fig. 7:**
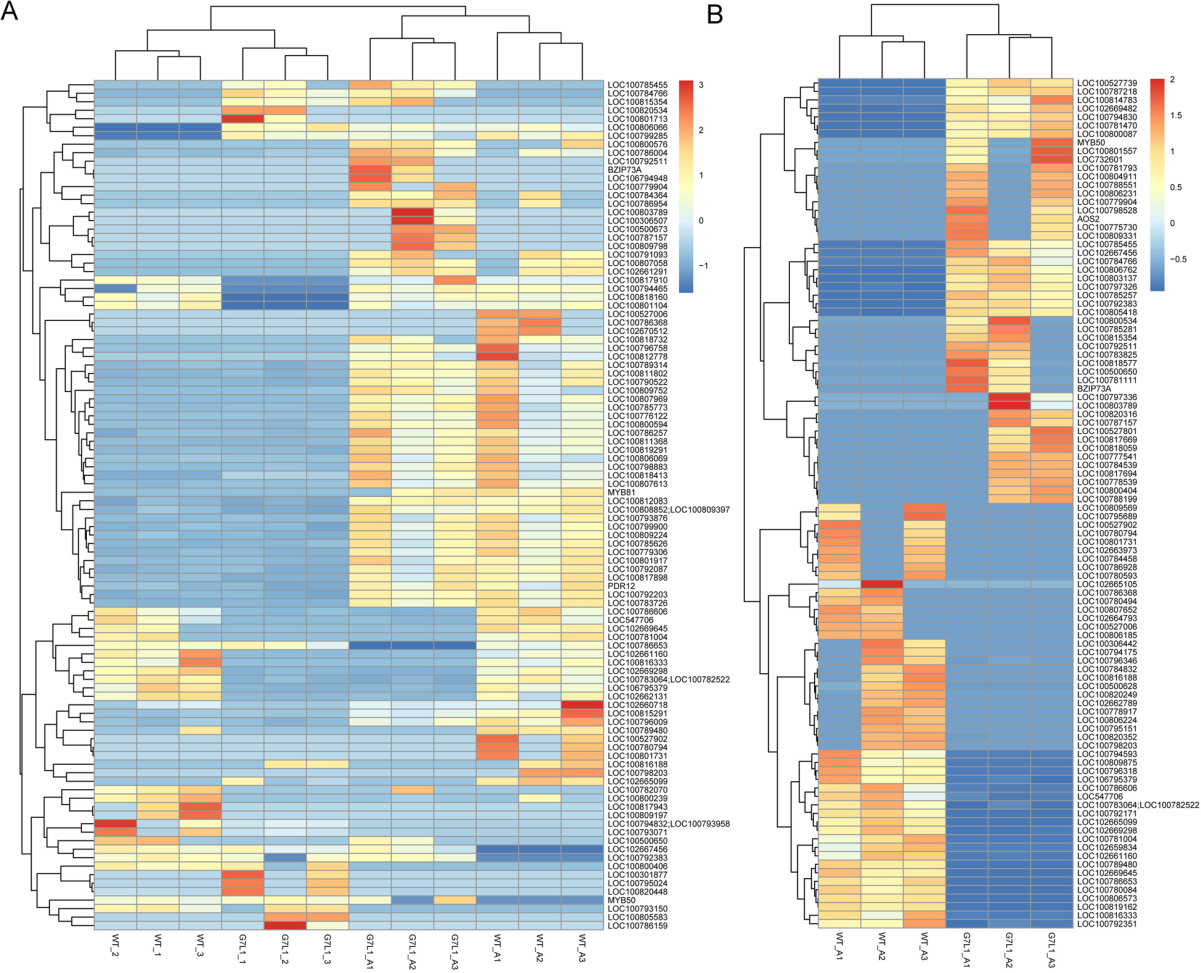
Cluster analysis of expression levels for differential genes. Log10 (FPKM + 1) was used for gene expression display. The horizontal axis was for the samples, while the vertical axis was for the genes. Different colors represented expression levels of different genes. The colors from blue to white to red represented the expression from lower to higher levels. Higher expression genes are marked in red and lower expression genes are marked in dark blue

**Fig. 8:**
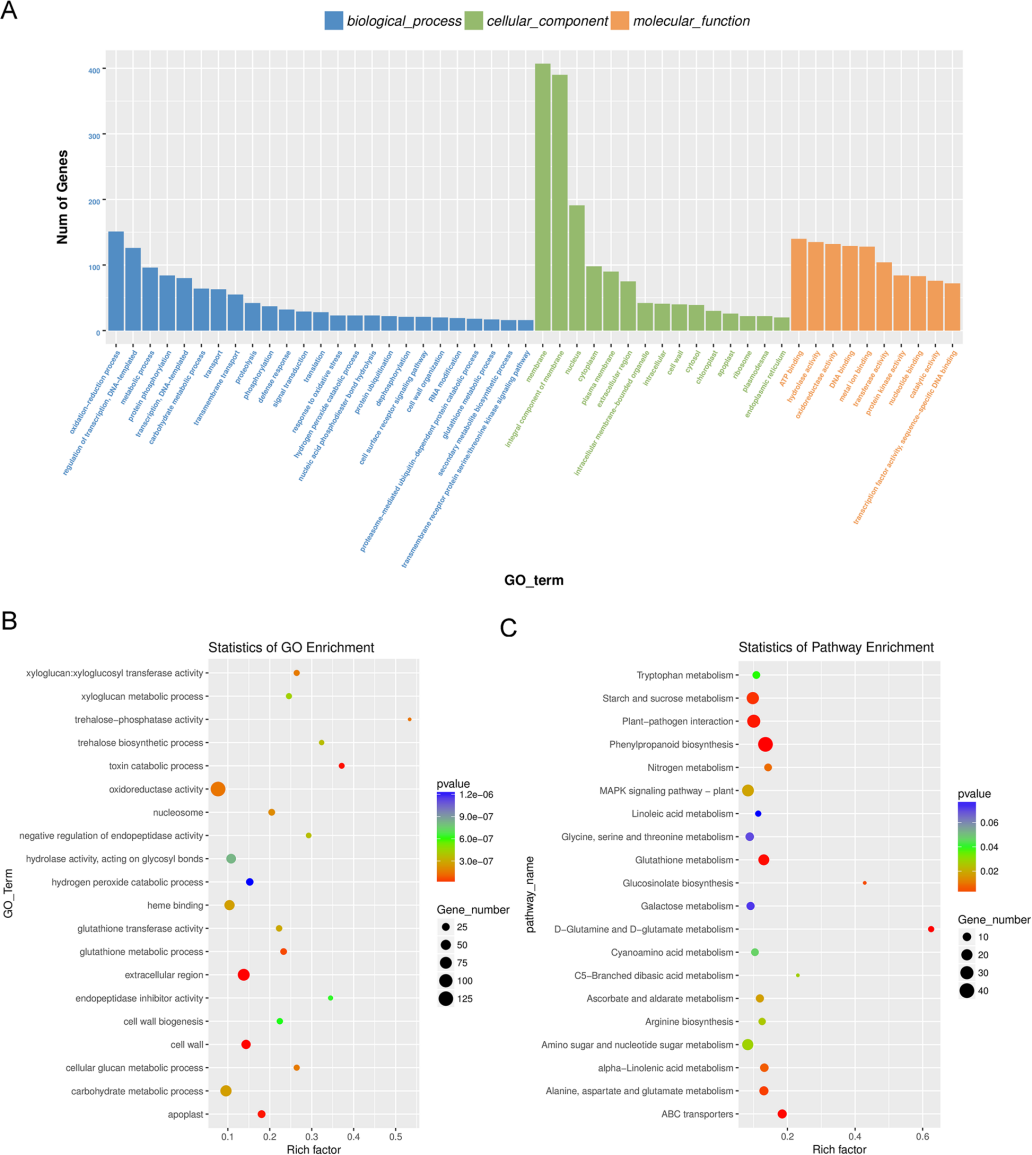
Transcriptome study of differentially expressed genes (DEGs) in four groups of items. **A** The histogram of GO enrichment analysis results. **B** GO enrichment scatter plot of differential genes. **C** KEGG enrichment scatter plot of differential genes. The results of GO enrichment analysis were shown in a bar chart with the three basic GO classifiers (Biological Process, Cellular Component, and Molecular Function) and the next level term of each type on the horizontal axis. The ordinate is the number of genes in a term (the term and its offspring). The abscissa of the scatter plot is the enrichment factor “Rich factor” which is the number of differential genes located in this KEGG (GO)/total genes located in this KEGG (GO). The vertical axis is the pathway term with high enrichment degree. After multiple checks, *P* value ranges [0, 1] were represented by colors. The size of the dot indicated the numbers of different genes in the term

In addition, the gene clustering analysis and the enrichment analysis based on gene ontology (GO) were carried out to investigate the pathways and metabolic processes regulated by the *GsMYB7* gene (Fig. 7 and 8). The results showed that the regulated genes were involved in the oxidation reduction process, regulation of transcription and metabolic process. Among these differential genes, about 410 genes were membrane-related, of which about 390 genes were integral component of membrane and about 190 genes were nuclear-related. These differential genes mainly encode transferase related proteins, metal ion binding related proteins, hydrolase related proteins and oxidoreductase related proteins (Fig. 8A, B). KEGG enrichment analysis showed that these genes are mainly involved in unsaturated fatty acid biosynthesis, cysteine and methionine pathways, protein processing in endoplasmic reticulum, and alpha-linolenic acid metabolism in plants (Fig. 8C).

**Fig. 9:**
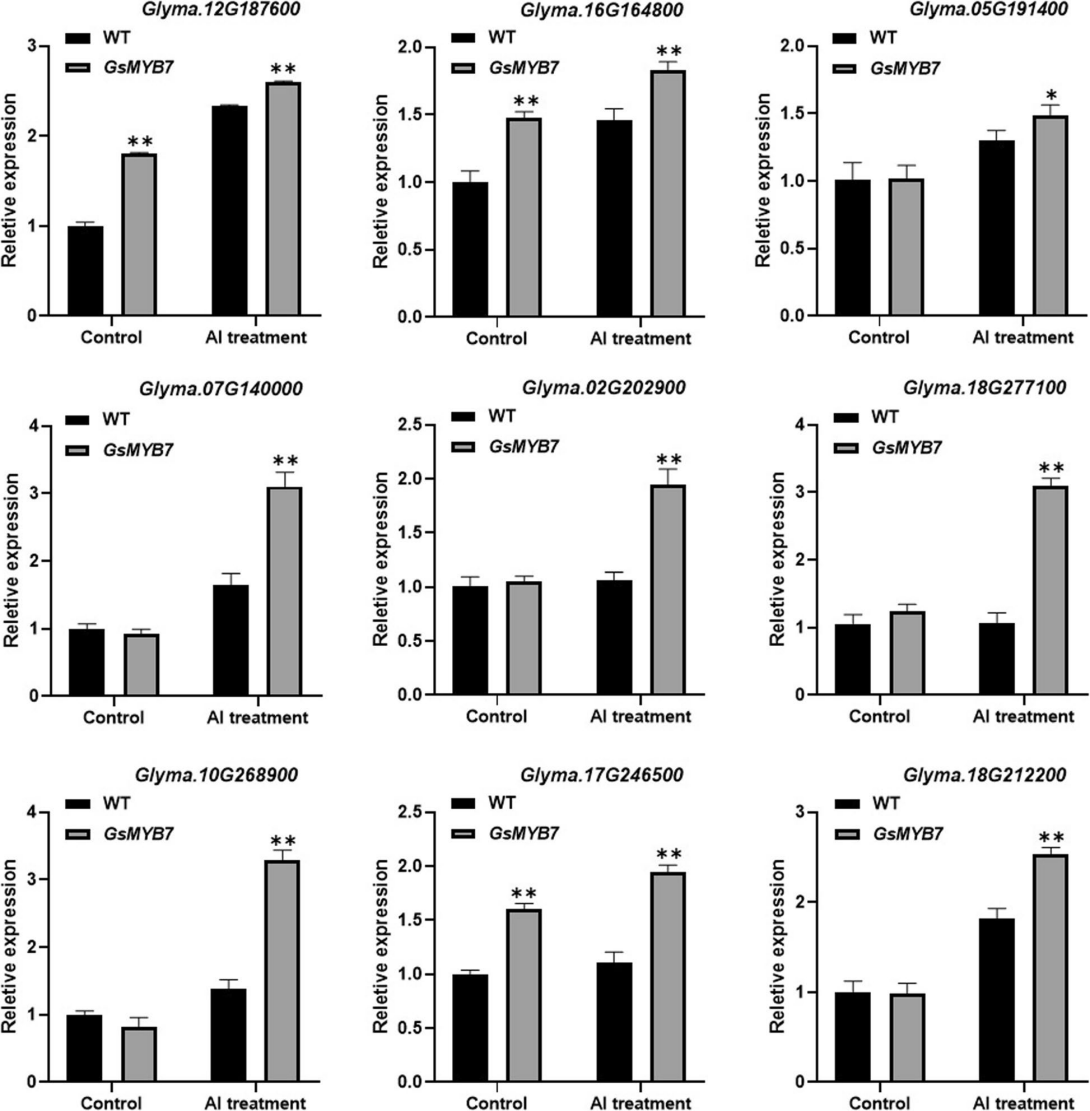
Expression patterns of downstream genes regulated by *GsMYB7*. Soybean plants were cultured in CaCl_2_ solution supplemented with 15 μM AlCl_3_ (pH4.5) for 8 h. The expression patterns of downstream responsive genes regulated by *GsMYB7* were detected. Using soybean *Actin3* as the internal reference gene, qRT-PCR was used to detect the gene expression level before and after aluminum treatment. The asterisks indicated that the transcripts of investigated genes in *GsMYB7* transgenic lines were significantly different from those of wild type treated with and without AlCl_3_ (*P < 0.05; **P < 0.01). All the information of gene sequences were from the NCBI database (https://www.ncbi.nlm.nih.gov/) shown in Additional file 1: Table S5

Furthermore, bioinformatics analysis was carried out on the significantly up-regulated genes in WT_AVSG7L1_A group, and 9 candidate genes were screened by combining the characteristics of R2R3-MYB TF DNA domain with the specific sequence of regulatory gene promoter. Finally, the expression of these 9 candidate genes through qRT-PCR validation was found to be basically consistent with the RNA-seq data. The candidate genes (*LOC100818413*, *LOC100817436、AOS2*, *LOC100786004*, *LOC547578*, *LOC100781111*, *BZIP73A*, *LOC100803789* and *LOC100803787*) were all up-regulated by acidic aluminum stress. After treatment with 15 μM AlCl_3_ for 8 h, the transcripts of these genes in *GsMYB7* transgenic soybean were significantly higher than those of wild-type HC6 (Fig. 9).

## Discussion

MYB TFs with four types are one of the large families in plants. Among them, R2R3-MYB TFs have two conserved DNA binding domains with a particularly alterable transactivation domain at the N-terminus or the C-terminus [10]. Each MYB repeat includes 50 to 53 amino acid and has 3 regularly distributed phenylalanine or tryptophan residues [30]. In this study, GsMYB7 protein had two conserved DNA binding domains which were located between 10–60 and 70–120 amino acid residues (Fig. 1A). It was localized in the nucleus (Fig. 2B) and maintained transcriptional activation in yeast cells (Fig. 2A). The results indicated that GsMYB7 protein has the characteristics of a R2R3-MYB TF.

It was well known that MYB family genes play a variety of roles in plant growth and development, and in plant abiotic stress since the *GL1* gene was first investigated in plants which is related to the development of epidermal hair [31]. In recent years, more reports are focused on the roles of R2R3-MYB TFs in abiotic stress. In *Arabidopsis*, overexpression of *AtMYB44* significantly improved salt tolerance and drought tolerance [32]. Overexpression of *MtMYBS1* in *Arabidopsis* reduced the damage to root growth caused by salt stress and osmotic stress, and increased the sensitivity of plants to ABA. It also enhances the transcription of the proline-synthesizing gene *P5CS* [33].Under high temperature stress, the activity of *AtMYB68* gene was significantly enhanced in *Arabidopsis* roots [34]. Overexpression of *OsMYB55* can enhance the resistance to high temperature by enhancing amino acid metabolism in rice [35]. Overexpression of *TaMYB30-B* can change the expression level of some drought-responsive genes in wheat, thus changing the physiological indexes of the plant and reducing the damage caused by high temperature stress [30]. The *RmMYB108* gene responds to low temperature stress in *Rosa multiflora* and endorse transgenic *Arabidopsis* with cold tolerance by reducing plant damage and promoting plant growth [36]. In this study, *GsMYB7* was induced by acidic aluminum stress (Fig. 3). The GsMYB7 protein was more similar to GmMYBJ3, GmMYBJ2 and GmMYB60 of the R2R3 subfamily proteins (Fig. 1B). It has been reported that MYB60 is the first TF involved in the regulation of stomatal movement. In tomato, *MYB60* is rich in leaves, and its expression is inhibited by abscisic acid. In *Arabidopsis* and grape, *MYB60* may be involved in the response to water stress [37–39]. *GmMYBJ3* is involved in the biosynthesis of soybean isoflavones by regulating *CHS8* and *CHI1A*, a secondary metabolite that plays a variety of roles in plant-microbial interaction and plant defense against abiotic stresses [40]. The results suggested that the GsMYB7 protein may play certain roles in soybean development and/or the tolerance to abiotic stresses.

Promoter analysis of R2R3-MYB family genes in *Arabidopsis*, soybean, maize, poplar and hypericum perforatum showed that most of the R2R3-MYB genes contained cis-acting elements related to abiotic stress in the regions of gene promoter. For example, previous studies have found that there are multiple stress-related elements such as TC-Rich (Defense and Stress Response Element), ABRE (ABA Response Element), WUN (Wound-Response Element) and TGA (Auxin Response Element) in the promoter of *SlMYB102* which is up-regulated by salt stress [41]. Further analysis found that 82% of the genes contained HSE elements involved in heat stress response in the promoter region. In addition, there are many stress elements in the promoter region of R2R3-MYB gene in plants such as ABRE element, MBS element, and CGTCA-motif/TGACG-motif. The R2R3-MYB genes have the functions of responding to abiotic stress [42]. In this study, multiple cis-acting elements related to light, ethylene and auxin were found in the 1500 bp nucleotide sequence upstream of the *GsMYB7* start codon. The *GsMYB7* gene with a constitutive expression pattern was induced by aluminum stress and rich in roots under the treatments with and without AlCl_3_ (Fig. 3). Overexpression of *GsMYB7* enhanced the tolerance of soybean to aluminum stress (Fig. 5). Previous studies indicated that *GmMYB81* upregulated by drought, salt and cold stress significantly increased seed germination rate and green seedling rate under salt and drought stress. GmMYB81 interacted with GmSGF14L which was an abiotic stress regulator to synergically enhance the tolerance of plants to abiotic stress [43]. The results suggested that the *GsMYB7* gene might play potential roles in abiotic stress and/or heavy metal stress (Additional file 1: Table S2).

The R2R3-MYB TFs play roles in response to abiotic stress by regulating the expression levels of downstream genes through certain pathways. For examples, overexpression of *GmMYB14* regulated plant structure through the brassinolide pathway to improve soybean yield and tolerance to drought stress [44]. In previous study, nine candidate genes were directly or indirectly regulated by *GsMYB7* under the treatments with and without AlCl_3_ using RNA-seq technology. The specific elements combined by R2R3-MYB TFs were analyzed using the regulatory genes’ promoters (Additional file 1: Table S5). Among them, *Glyma.12G187600*, *Glyma.16G164800*, *Glyma.07G140000*, *Glyma.02G202900* and *AOS2* (*Glyma.17G246500*) contain the core sequence TAACTG at the upstream regions less than -2000 bp of the promoters which can be bound specifically by MYB TFs. AtMYB20 binds to the promoter region containing the MYB recognition sequence (TAACTG) and enhances salt tolerance of transgenic plants by down-regulating the expression of negative regulators in ABA signal transduction [45]. ZmMYB14, a typical R2R3-MYB TF, binds to the -280 ~ -151 bp region of maize *ZmBT1* promoter and promotes its activity through the TAACTG site to regulate maize starch biosynthesis [46]. The qRT-PCR results showed that the genes of *Glyma.12G187600*, *Glyma.16G164800* and *AOS2* (*Glyma.17G246500*) were significantly upregulated by *GsMYB7* with or without the treatments of AlCl_3_ than those of wild type (Fig. 9). Previous studies have shown that tobacco plants with overexpression of *AOS2* (*Glyma.17G246500*) has higher resistance to cotton bollworm [47]. The *Glyma.16G164800* gene encodes an ERF TF related to abiotic stress in plants [48]. *GmERF5* was significantly induced and its promoter activity was upregulated under ethylene (ET), abscisic acid (ABA), Phytophtophella, salt and drought treatments. *GmERF5* was not only involved in the induced defense response, but also involved in the ABA-mediated salt and drought tolerance pathway [49]. In present study, some genes such as *Glyma.05G191400*, *Glyma.07G140000*, *Glyma.02G202900*, *BZIP73A* (*Glyma.18G277100*), *Glyma.10G268900* and *Glyma.18G212200* were significantly up-regulated in *GsMYB7* transgenic soybean plants after aluminum treatment (Fig. 9). Among them, *BZIP73A* is described to encode a BZIP transcription factor, *Glyma.18g212200* encodes a protein kinase that interacts with CBL. Previous studies have shown that calcineurin B-like protein (CBL) and its interaction protein kinase (CIPK) play an important role in response to abiotic stress. *TaCIPK27* overexpression enhanced drought tolerance through an ABA-dependent pathway [50]. The results suggested that overexpression of *GsMYB7* in soybean could synergistically improve the tolerance of transgenic plants to acidic aluminum stress by regulating the downstream genes.

## Conclusions

The *GsMYB7* gene from wild soybean encoding a R2R3-type MYB TF was upregulated by acidic aluminum stress, and rich in the roots with a constitutive expression pattern in soybean. *GsMYB7* overexpression could improve the tolerance of soybean to aluminum stress by alleviating the inhibition of acidic aluminum stress on soybean taproots and reducing the accumulation of Al^3+^ in root tips. The downstream genes regulated by *GsMYB7* under acidic aluminum stress were identified by means of RNA-Seq. The results expounded the molecular basis for further analysis of *GsMYB7* tolerance to acidic aluminum stress and provide a theoretical basis for soybean Al-resistant breeding.

## Methods and materials

### Plant materials and growth conditions

The seeds of soybean cultivar HC6 and BW69 (*Glycine soja*) were obtained from the Guangdong Subcenter of the National Center for Soybean Improvement (Guangzhou, China).

Seeds were germinated in sterilized vermiculite with room temperature set at 28/26 ◦C and the light time set as 14 h-light/10 h-dark under a light intensity of 110 µmol/(m^2^·s) [51]. The BW69 seeds cut gently on the back with a single side blade were germinated in sterilized vermiculite. When the seedling cotyledons unfeld, the seedlings were pulled out and washed with deionized water. After pre-cultured in nutrient solution for 48 h and treated with aluminum solution, they were then moved into the 0.5 mM simple calcium solutions (pH 4.5) containing 0, 15, 30, 50, 75 and 100 μM AlCl_3_, respectively [52]. The experiments were designed with 3 repetitions using 20 seedlings per treatment for individual replicate. The samples of seedlings were taken from the roots which were then freezed in liquid nitrogen and stored at -80℃.

### Isolation and bioinformatics analysis of the *GsMYB7* gene

The *GsMYB7* gene from *Glycine soja* BW69 line were up-regulated in the expression profile of acid-resistant aluminum genes, and the login number on the website of the National Center for Biotechnology Information (NCBI) was XP_003527079.1 using the NCBI database for searching for the sequence information of the candidate gene. The software DNAMAN9.0 was used for sequence alignment [53], while the phylogenetic tree was constructed using the proximity method (NJ) [54] which was completed by the software MAGE 7.0 [55]. The root total RNA of *Glycine soja* BW69 line extracted by *Tri*zol solution (Vazyme Biotech Co., Ltd., Nanjing, China) was reverse-transcribed into cDNA (HiScript® III RT SuperMix for qPCR, Vazyme Biotech Co., Ltd.; Nanjing, China). The full-length of *GsMYB7* gene was cloned by using specific primers and high-fidelity DNA polymerase (Phanta Max, Vazyme Biotech Co., Ltd.; Nanjing, China) (Additional file 1: Table S3). Total RNA extraction, cDNA generation and RT-PCR amplification were performed according to the methods described in detail previously [56]. The PCR products were detected with 1% agarose gel electrophoresis (GenStar Kit, Genstar Development Company, Calgary, AB, Canada), and the purified PCR products were inserted into the polyclonal sites of zero-background cloning pLB vector to form the GsMYB7-pLB construct (Tiangen Lethal Based Fast Cloning Kit, Beijing, China) [57]. The fusion vector was then transformed into *Escherichia coli* DH5α. The positive clones of *GsMYB7* gene were then identified by PCR and enzyme digestion, and the CDS sequence of *GsMYB7* gene was obtained by sequencing identification [Sangon Biotech (Shanghai) Co., Ltd., China] [3, 51].

### Gene expression analysis by quantitative real time PCR (qRT-PCR)

The total RNA of samples was extracted from plant seedlings using *Tri*zol solution (Vazyme Biotech Co., Ltd., Nanjing, China). The complementary DNA (cDNA) templates were synthesized using the HiScriptII first strand cDNA synthesis kit (Vazyme Biotech Co., Ltd., Nanjing, China) [58]. Using SYBR PremixEx-Taq^TM^II (Takara Bio Inc., Kusatsu, Japan), the RNA transcripts of the relative genes were measured using a real-time CFX96TM system (Bio-Rad, Hercules, CA, USA) for qRT-PCR [51]. The following procedure was performed for qRT-PCR: 94 ◦C for 3 min; 39 cycles of denaturation at 94 ◦C for 10 s, annealing at 57 ◦C for 10 s, elongation at 72 ◦C for 30 s. The reference gene was *Actin-3* in soybean. Finally, the relative expression values were calculated using the comparative cycle threshold method 2^−△△ct^. The experiments were carried out with three independent organisms [25].

### Subcellular localization of GsMYB7 protein

The subcellular localization of GsMYB7 protein was analyzed using the method described by Tamara et al.and and Yan et al.[59, 60]. The GsMYB7 protein subcellular localization was predicted using a web server Cell-PLoc 2.0 (http://www.csbio.sjtu.edu.cn/bioinf/Cell-PLoc-2/). The recombinant fragment containing the CDS sequence of *GsMYB7* gene (without termination codon) was amplified and purified with the restriction sites of *Nco*I and *Spe*I at both ends (Additional file 1: Table S3). The expression vector pCAMBIA1302-eGFP was linearized with *Nco*I and *Spe*I restriction enzymes, and the recombinant fragment was ligated to construct the fusion expression vector pCAMBIA1302-eGFP-GsMYB7. The plasmids of pCAMBIA1302-eGFP and pCAMBIA1302-eGFP-GsMYB7 were transformed into the cells of *Agrobacterium tumefaciens* GV3101, respectively. The transformed cells of GV3101 and viral protein P19 were resuspended in the prepared osmotic buffer (The main components are 10 mM MgCl_2_, 10 mM MES- KOH and 100 μM acetyleugenone) for culture and then mixed in the same volume. After sitting at room temperature for 3 h, the bacterial fluid was injected into the lower epidermis cells of 4-week-old leaf in *Nicotiana tabacum*, and the transformed leaves after being cultured for 48 h were observed by laser confocal microscopy (Carl Zeiss, Jena, Germany).

### Transactivation assay

Transcriptional activation assay of *GsMYB7* was analyzed using the method described by Ma et al. [61]. To verify the activation activity of GsMYB7 protein, the full-length sequence of *GsMYB7* was inserted into the sites of BamHI and EcoRI restriction enzymes of pGBKT7 vector to form the fusion expression vector pGBKT7-GsMYB7. The constructed vector pGBKT7-GsMYB7 and the empty vector pGBKT7 were then transformed into yeast Y2H strain, respectively. The positive transformants were cultured on SD medium which was lacking Trp (SD/-Trp) at 30 ℃ for 2 or 3 days. The positive colonies were transferred from SD/-Trp medium to chromogenic SD/-Trp medium containing 200 μg X-α-gal. After cultured for 3 days, the status of colony growth could be detected on selective medium.

### Construction of *GsMYB7* expression vector and genetic transformation of soybean

The CDS sequence of *GsMYB7* amplified by PCR was inserted between the restriction sites of *Bam*HI and *Kpn*I from the modified intermediate vector pUC18 to construct the intermediate vector pUC18-GsMYB7. The pUC18-GsMYB7 was digested with a *Hin*dIII enzyme to recover the target fragment of 35S-GsMYB7-NOS. The DNA fragment containing the target gene was inserted into the *Hin*dIII site of pZY101 vector to construct GsMYB7-pZY101 vector which was then transformed into the competent cells of *Escherichia coli* DH5α. The positive clones of pZY101-GsMYB7 were identified by bacterial liquid PCR, enzyme digestion and DNA sequencing. The expression plasmid of pZY101-GsMYB7 was transformed into *Agrobacterium tumefaciens* EHA101 and then used for soybean genetic transformation. The soybean HC6 was used as receptor for *GsMYB7* genetic transformation using the cotyledonary node method described by Zeng et al. [62]. The obtained transgenic plants were identified by herbicide tolerance, DNA and RNA analysis. T_3_ transgenic positive lines of *GsMYB7* were further used to analyze the expression pattern response to aluminum stress, the phenotypes tolerant to aluminum stress, and molecular identification, etc. [63]. The primer information used in this experiment was shown in Additional file 1: Table S3.

### Phenotype analysis of soybean root growth

The full seeds of *GsMYB7* transgenic lines and wild type (WT) for the phenotype analysis to aluminum stress were sterilized with 1.0% (v/v) NaClO for 5 min and germinated in sterilized moist vermiculite sand which was placed under a condition of photoperiod of 14-h day/10-h night and 28 /25℃ (day/night) temperature circulations for 3 or 4 days in light incubator. The soybean seedlings cultured in 0.5 mM CaCl_2_ (pH4.5) solution for 24 h were transferred to nutrition barrels separately which contained different concentrations of AlCl_3_ (0 and 25 μM) for 48 h. The data were analyzed by using the software Image J and Root Scanner [64].

### Determination of Al^3+^ content under acidic aluminum stress

The seeds of *GsMYB7* transgenic lines and WT were germinated in darkness for 3 days and then transferred to Hoagland solution under light incubator 16 h (26◦C)/8 h (24◦C) day/night. After cultured for 3 days, the seedlings with relatively consistent length of taproots were chosen and immediately transferred to Hoagland solution with 25 µM AlCl_3_ (pH4.5) for 24 h [65]. The 0–2 cm root segments of seedlings were cut and washed three times with distilled water. The content of Al in the root segments extracted by 2 M HCl for 48 h was determined by inductively coupled plasma atomic emission spectrometry (ICP-AES, IRIS-Advantage, Thermo Elemental, Waltham, MA, USA) [66].

### Transcriptome sample preparation and analysis

To determine the downstream genes and regulatory pathways regulated by TF GsMYB7 under acidic aluminum stress, the RNA-seq technology was used to find the differentially expressed transcriptome of *GsMYB7* transgenic soybean and HC6. The root samples were taken from the seedlings of *GsMYB7* transgenic plants and HC6 treated with or without 25 µM AlCl_3_ (pH4.5, 0.5 mM CaCl_2_). After the treatment for 8 h, the samples of 2 cm root tip were taken for transcriptome sequencing analysis [67]. Each biological replicate consisted of three pieces of samples for the same treatment, which were sent to Hangzhou Lianchuan Biotechnology Co., Ltd (Hangzhou, China) for RNA sequencing analysis (Additional file 2: Table S6-9). The basic process of in-depth analysis of transcriptome sequencing data is to use Tophat comparison results to assemble transcripts, calculate the abundance of these transcripts, and detect the differential expression between the samples. Statistical analysis and graphical display of the final data to form the RNA sequencing results including differential gene expression heat map, scatter plot and principal component analysis map, etc. [57].

### Statistical analyses

All experiments were repeated at least three times independently, with tissue samples from 20 plants in each experiment. The data were the mean ± SD. The SPSS Statistics (v.22) and GraphPad Prism 8 softwares were used to analyze and process the data. Differences were compared using Student’s t-test. The statistical significance of the difference for the data was indicated by an asterisk (*P < 0.05; **P < 0.01) [25].

## Supplementary Information

**Additional file 1:** **Table S1.** Sequence information of *GsMYB7*. **Table S2.** Primers used in this study. **Table S3.** A cis-acting element in the upstream nucleotide sequence of the initiation codon of *GsMYB7*. **Table S4.** The information of *MYB* genes from soybean. **Table S5.** *GsMYB7* downstream candidate gene information.

**Additional file 2:** **Table S6.** G7L1VSG7L1_A_Gene_differential_expression. **Table S7. **WT_AVSG7L1_A_Gene_differential_expression. **Table S8.** WTVSG7L1_Gene_differential_expression. **Table S9.** WTVSWT_A_Gene_differential_expression.

**Additional file 3:** **Table S10.** Bar primers were used to identify the transgenic lines by PCR.

## Data Availability

The datasets generated for this project can be found in the All raw sequences available in the NCBI short-read archive under accession number PRJNA812620 (https://www.ncbi.nlm.nih.gov/).

## Abbreviations

HC6: The soybean cultivar Huachun 6
WT: Wild type
qRT-PCR: Quantitative real time PCR
Y_2_H: Yeast two-hybrid
