# Supplementary material for: GsMYB7 encoding a R2R3-type MYB transcription factor enhances the tolerance to aluminum stress in soybean (Glycine max L.)
Source: BMC Genomics. 2022 Jul 22;23:529. doi: 10.1186/s12864-022-08744-w (PMC9306046; doi:10.1186/s12864-022-08744-w)
Supplement: Supplementary file 1 — Additional file 1: Table S1. Sequence information of GsMYB7. Table S2. Primers used in this study. Table S3. A cis-acting element in the upstream nucleotide sequence of the initiation codon of GsMYB7. Table S4. The information of MYB genes from soybean. Table S5. GsMYB7 downstream candidate gene information. [file 12864_2022_8744_MOESM1_ESM.docx]

**Table S1. Sequence information of *GsMYB7***

**Transcript sequence information of *GsMYB7***

ATAGCCCCAGTTGCAGAACCTTGTCTCTGCATCATCATAATTCTCTCAATTGAGGAGCCCAAAGGGATCAAATCTCTTCAACCCAGAAGAAGAAAAACAAAAGAGAAATTATAGAATTAAGTTGCTATAGCTAATAGCTATAACTGGCTTAGATATACCATTTTTATTTTATTTTTATTTTGGGGTGTACAAAATGGGAAGACCACCTTGCTGTGATAAAATTGGGATTAAGAAAGGGCCTTGGACTCCTGAGGAAGACATCATCTTGGTCTCTTACATTCAAGAACATGGACCCGGAAATTGGAGATCGGTTCCCAGTAACACAGGTTTGATGAGATGCAGCAAAAGCTGCAGACTCAGATGGACCAACTATCTCCGACCTGGTATCAAACGAGGCAATTTCACCGATCATGAAGAGAAAATGATAATCCACCTCCAAGCTCTTTTGGGTAACAGATGGGCTGCTATAGCTTCCTACCTTCCACAAAGGACAGACAATGACATAAAGAACTATTGGAACACCCATTTGAAGAAGAAGCTGAAGAAGATGCAAATTGGGGGTGGTAGTGATGATGATAATAATGATGACAAATCAAACTCTTCTAACAATTCACAAATAAAGGGTCAATGGGAAAGAAGACTTCAAACAGATATCCACATGGCCAAACAAGCCTTATGTGAGGCCCTATCTCTTGACAAACCAACCCAAATTTTCCCAGAGACCAAATTACCCTCCACTTCTTCACACCACCACCCCACAACAACAACAACACCAAACCAAACAACATCCTTGTATGCATCAAGCACAGAAAACATAGCCAGATTGTTGGAGAATTGGATGAAGAAATCACCAAATATGACGACCACGACGACAACAACAATGGAGACAAAACCCTTCAGCAATAATAACATGGTAATAACCACAGGGTCTAGTTCTAGTGAGGGAACACAAAGCACAATCACATGCACACAGGAGTATGCCCTTGACTCCTTGTGGAGCTTCAACTCTGAACGCTCTTCTCAATCTGAAGAAAACACCAACTTGGGTGAGAGCAAGCCACAGTACCAAGAGCCTCAAGAGACACAAGTCCCTCTCATGTTGCTGGAGAATTGGCTCTTTGATGATGCTGCACCTCAATGCAATGAAGATCTAATGAACATGTCACTCGAGGAAAGTACAGAAGGGTTGTTCTAATTGACGATCCTAACGATGTAGCGGACTTGAGGTCGGAACAAAATTCATGTAACCAAATCAACCACAAAGGGTCTTTGAAAAAAAAAAATAAGAGTATGCGTGCGTGTGTGAATTAAACTTTATTAGAGTTCTTGCAAAATTGAACTACTTTTCTAGGCCCCCCAGTAGGAAGGTAACTCCCTCAATACAAGCAAAGTGTACATTACAAGTACATCTATAATTAACTACGATTATGTTAGCTTATCTTATCTACCATAATAACTGGAGTTGAATATATGTTGTGTTTTTACTTT

**CDS sequencing** **information of *GsMYB7***

ATGGGAAGACCACCTTGCTGTGATAAAATTGGGATTAAGAAAGGGCCTTGGACTCCTGAGGAAGACATCATCTTGGTCTCTTACATTCAAGAACATGGACCCGGAAATTGGAGATCGGTTCCCAGTAACACAGGTTTGATGAGATGCAGCAAAAGCTGCAGACTCAGATGGACCAACTATCTCCGACCTGGTATCAAACGAGGCAATTTCACCGATCATGAAGAGAAAATGATAATCCACCTCCAAGCTCTTTTGGGTAACAGATGGGCTGCTATAGCTTCCTACCTTCCACAAAGGACAGACAATGACATAAAGAACTATTGGAACACCCATTTGAAGAAGAAGCTGAAGAAGATGCAAATTGGGGGTGGTAGTGATGATGATAATAATGATGACAAATCAAACTCTTCTAACAATTCACAAATAAAGGGTCAATGGGAAAGAAGACTTCAAACAGATATCCACATGGCCAAACAAGCCTTATGTGAGGCCCTATCTCTTGACAAACCAACCCAAATTTTCCCAGAGACCAAATTACCCTCCACTTCTTCACACCACCACCCCACAACAACAACAACACCAAACCAAACAACATCCTTGTATGCATCAAGCACAGAAAACATAGCCAGATTGTTGGAGAATTGGATGAAGAAATCACCAAATATGACGACCACGACGACAACAACAATGGAGACAAAACCCTTCAGCAATAATAACATGGTAATAACCACAGGGTCTAGTTCTAGTGAGGGAACACAAAGCACAATCACATGCACACAGGAGTATGCCCTTGACTCCTTGTGGAGCTTCAACTCTGAACGCTCTTCTCAATCTGAAGAAAACACCAACTTGGGTGAGAGCAAGCCACAGTACCAAGAGCCTCAAGAGACACAAGTCCCTCTCATGTTGCTGGAGAATTGGCTCTTTGATGATGCTGCACCTCAATGCAATGAAGATCTAATGAACATGTCACTCGAGGAAAGTACAGAAGGGTTGTTCTAA

**Amino acid sequence of GsMYB7 protein**

MGRPPCCDKIGIKKGPWTPEEDIILVSYIQEHGPGNWRSVPSNTGLMRCSKSCRLRWTNYLRPGIKRGNFTDHEEKMIIHLQALLGNRWAAIASYLPQRTDNDIKNYWNTHLKKKLKKMQIGGGSDDDNNDDKSNSSNNSQIKGQWERRLQTDIHMAKQALCEALSLDKPTQIFPETKLPSTSSHHHPTTTTTPNQTTSLYASSTENIARLLENWMKKSPNMTTTTTTTMETKPFSNNNMVITTGSSSSEGTQSTITCTQEYALDSLWSFNSERSSQSEENTNLGESKPQYQEPQETQVPLMLLENWLFDDAAPQCNEDLMNMSLEESTEGLF*

***GsMYB7* transcriptional initiation of ATG upstream 1500bp sequence**

TACTTTCTCTGTTCTTTTTTATTTGTCTTTTAAGGTTATTTTGTAAAAATTAAAAAATATAATAATTTTTTTAATTTTAATAAAATAGCTGTGAAGTTTTTTATTTTTACTTCTTTTTCCTTCCACTCTACAATAAATACATGAATAAACTAATTAAAGATTATGAGATAAGTGAAAATTTAATTAATAAAATAATAATTAATGTGATCTTAGCAATAGATAAATATAGAAAAAAACTTATAAAAAAATTACACAATTAAAAAAAGAAACGAGGTAATATACTGTAATTGTTATTACTCCATTTTATCTTAAGGAAATGGCTAAAGTTTCTTACAAATTTTTACTATTTCATAAAAAAAAAACATAAAATGAAAAGTTAAATACATTTAACATTATTTTTTAAAAGAAATACAAAGAAAAGAAAGTAGAATAATTAATACAAGAATGTCGTTATTTTAATAACTATAGCTTTATTTTGATAATTTTCATAATTAATTAAAATTAAATAATATATCGGATGATCAAGTAGTTTACGATTAAAATCTTGTTGATTATTAAATATGGAATAATTCGTGTTAGAAAAAACAATATTCACATTGTGTGTGTTAAGATTAAGATGAATCATCACTTTTGGCATAGGCTCTTTCATATAAATAGCTTGAAAAAAATTTAAATATATTTTTAATTCTTATAATTTAATATTTTTTTATTTTTTATTCTTGCAAAATTATTTTTTTACTTTTATTCCATATAAATGCAAATTATGTATATTTTATTTTTTTTTTCCTTAGAGCGCATTAGATAATATTTTAAATATTAAAAAAATGCTACTAAATTTTATAAGGAAGAAAAATAAAAAAAAATTTGCAAGTACAACAAAAAATATAATTTTGCAATGACAAAATTTTTTTTTTAATTATAAGAACTAAAAATTATATTTAAAAAAATACAGACTTTACAGTAACATTAACTACTAAAATTATTTCAAAGACTTTTTGTTTTTGGATTGTAAAACACCTATTTTTCCTCCGGGATTTGCTTTGCTTCTGGGTGTTTCCACTGTTCCCATAATAACAATAATGAATGCTGCAGCACGGATTTGTGTGAAAGGTTAATGTAGAAAGAGAGTGAAGGGGTGGACGCATAGCTGACTTTTTGCAATGCACGAGTAAATGCCTTTCTTGTCTGTTTTGACTCCTTTTTTTTTTTTTTCTTTCTTTCTTCATTTACTTACCTCATTTTTTCTCTCTAGCTAGTCAGCGAATCAACCATTAATCTTCTATTTCCTCTAATTCTCTTCTCTCTCTATATATATGTATCTGCCTCATGGAAAATAGACAACATAGCCCCAGTTGCAGAACCTCTCTCTGCATCATATTCTCAATTGAGGAGCCCAAAGGTCAAATCTTCAACGAAGAAGAAGAAAACAAAAGAAGAAGAAGAATAATAAGAATAATTATAGTGTTAGTTTTTTTTTTTTTTTTTTGTGTGATAATAATA

**Table S2. A cis-acting element in the upstream nucleotide sequence of the initiation codon of *GsMYB7***

| ***Cis*-element** | **Sequence(5’-3’)** | **Position** | **Function** |
| --- | --- | --- | --- |
| TATA-box | TATA | -38 (-); -508 (+); -237(+); -674(+); -224(+); -668(+); -277(+); -685 (-); -57(+); -647(+); -238(+); -673(+); -236(+); -665 (-); -463(+); -688 (-); -509(+); -154(+); -689(+);-648(+); -748(+); -764 (-); -808 (-); -835 (-); -882(+); 915(+); -931(+); 1306(-); -1455(+); | core promoter element around -30 of transcription start |
| AE-box | AGAAACTT | -323 (-); | Part of a module for light response |
| AT1-motif | AATTATTTTTTATT | -851 (-); | Part of a light responsive module |
| Box 4 | TTTCAAA | -182 (+); -1270 (-); -432 (+); -197(+) ;-490(+); | part of a conserved DNA module involved in light responsiveness |
| CAAT-box | CAAT | -20 (-); -893 (+); -595 (-); -1381 (+); -286 (-); -1097 (-); -757 (+); -1401 (-); -213 (+); -1033 (-); -585 (+); -1383 (-); -333 (+); -1158 (+); -862 (-); -1005 (+); -130(+); 1074(+); -253(+); | common cis-acting element in promoter and enhancer regions |
| ERE | ATTTCAAA | -345(+); -806(+); | Ethylene-responsive element |
| GA-motif | ATAGATAA | -215(+) | part of a light responsive element |
| TCT-motif | TCTTAC | -328(+) | part of a light responsive element |
| [TGA-element](http://bioinformatics.psb.ugent.be/webtools/plantcare/cgi-bin/show_site_info.htpl?QWhere=ID_of_Site like 'AACGAC'&StartAt=0&NbRecs=10) | AACGAC | -446(-) | auxin-responsive element |

**Table S3. The primers used in the study**

| **Primer Name** | **Purpose** | **Forward Primer (5'-3')** | **Reverse Primer (5'-3')** |
| --- | --- | --- | --- |
| Actin3 | qRT-PCR | GCACCACCGGAGAGAAAATA | GTGCACAATTGATGGACCAG |
| GsMYB7 | qRT-PCR | GGACCAACTATCTCCGACCTG | GCCCATCTGTTACCCAAA |
| GsMYB7-GFP | Cloning processes in subcellular localization | aattctgcagtcgacggtaccAAATGGGAAGACCACCTTGCT | ccttgctcaccatcaggatccTGAACACCCTTCTGTACTTTCCTC |
| GsMYB7-GFP-JD | Detection processes in subcellular localization | TAAGGGATGACGCACAATC | TGCAGATGAACTTCAGGGT |
| *GsMYB7*-pGBKT7 | Cloning processes in transcriptional activation | aggacctgcatatggccatggAAATGGGAAGACCACCTTGCT | ccgctgcaggtcgacggatccTTTAGAACAACCCTTCTGTACTTTCC |
| *GsMYB7*-pGBKT7-JD | The detection process in transcriptional activation | ATCATGGAGGAGCAGAAG | GGGGTTATGCTAGTTATG |
| BKGmM7 | Overexpression of Huachun 6 experiment | GGATCCATGGGAAGACCACCTTGC | GGTACCTTAGAACAACCCTTCTGT |
| pZY101-*GsMYB7* | Overexpression test | GGCAGAGGCATCTTCAACG | GCTCACTCATTAGGCACCC |
| Bar-JD | Bar gene identification | AAGTCCAGCTGCCAGAAACC | AAGCACGGTCAACTTCCGTA |
| pMU103-*GsMYB7* | Interference vector fragment cloning | ATACTAGTGGCGCGCCGGGTGGTAGTGATG | CCGAGCTCGCCTAGGACTGTGGCTTGCTC |
| pMU103-*GsMYB7-*JD1 | Interference vector fragment detection | ATCTGAATAAGAGGGGAAAC | GTCCTTAAACACTCATCAGT |
| pMU103-*GsMYB7-*JD2 | Interference vector fragment detection | TATCATGCGATCATAGGCGT | AGGCTCACCAAACCTTAAAC |
| *Glyma.12g187600* | qRT-PCR | CAGCAGGTCTCCGTCGC | GAGGATCTGGAGCGTGAAGG |
| *Glyma.16g164800* | qRT-PCR | AAGGAGACTCATCATCAGTGACAG | TGGTGGGTTTTCAGTGACGG |
| *Glyma.05g191400* | qRT-PCR | ACGATCGCATCACCAAGACC | CACAATTCCTGCTGAAACCTCC |
| *Glyma.07g140000* | qRT-PCR | GCAGAATCCCTTGTGATTGTTGA | AGCACCCACAACCTTGTCAT |
| *Glyma.02g202900* | qRT-PCR | CCGTCGTTTATGGTGGTGGA | CAGGTGCGGAATTCTGGGAG |
| *Glyma.18g277100* | qRT-PCR | ACTCTTTGGACCTGCTTGGT | TCTCAAAAGGGTGGCAAAGAC |
| *Glyma.10g268900* | qRT-PCR | ACCCTAGGTCTCAGCCGATT | GAGCATGATAAGTCCGGCGA |
| *Glyma.17g246500* | qRT-PCR | TCCTCAACCAAACAACGCTCT | GGATTTTGCGGATCGGAAGC |
| *Glyma.13g338400* | qRT-PCR | TTTCCAGCAGAGGCATGAGG | TGCAACCCAGAAGTTTAGGCT |

**Table S4. The information of *MYB* genes from soybean**

| **Serial number** | **Gene** | **Gene ID** | **Locus tag** | **References** |
| --- | --- | --- | --- | --- |
| 1 | *GmMYB181* | 778046 | *GLYMA_16G073000* |  |
| 2 | *GmMYB14* | 100101836 | *GLYMA_15G259400* | Holl, J., Vannozzi, A., Czemmel, S., D'Onofrio, C., and Walker, A.R., et al. (2013). The R2R3-MYB transcription factors MYB14 and MYB15 regulate stilbene biosynthesis in Vitis vinifera. Plant Cell 25, 4135-4149. doi:10.1105/tpc.113.117127 |
| 3 | *GmMYB12B2* | 100819467 | *GLYMA_19G218800* | Li, X.W., Li, J.W., Zhai, Y., Zhao, Y., and Zhao, X., et al. (2013). A R2R3-MYB transcription factor, GmMYB12B2, affects the expression levels of flavonoid biosynthesis genes encoding key enzymes in transgenic Arabidopsis plants. Gene 532, 72-79. doi:10.1016/j.gene.2013.09.015 |
| 4 | *GmMYBJ1* | 100818077 | *GLYMA_05G051700* |  |
| 5 | *GmMYB84* | 778042 | *GLYMA_05G234600* | Zhang, W., Wang, N., Yang, J., Guo, H., and Liu, Z., et al. (2020). The salt-induced transcription factor GmMYB84 confers salinity tolerance in soybean. Plant Sci 291, 110326. doi:10.1016/j.plantsci.2019.110326 |
| 6 | *GmMYBJ3* | 100794319 | *GLYMA_06G193600* |  |
| 7 | *GmMYBZ2* | 780553 | *GLYMA_11G107100* |  |
| 8 | *GmMYB54* | 778030 | *GLYMA_12G032200* | Zhong, R., and Ye, Z.H. (2012). MYB46 and MYB83 bind to the SMRE sites and directly activate a suite of transcription factors and secondary wall biosynthetic genes. Plant Cell Physiol 53, 368-380. doi:10.1093/pcp/pcr185 |
| 9 | *GmMYB48* | 778031 | *GLYMA_06G003800* | Ashrafi-Dehkordi, E., Alemzadeh, A., Tanaka, N., and Razi, H. (2018). Meta-analysis of transcriptomic responses to biotic and abiotic stress in tomato. PeerJ 6, e4631. doi:10.7717/peerj.4631 |
| 10 | *GmMYB56* | 778033 | *GLYMA_06G160500* | Zhang, Y., Liang, W., Shi, J., Xu, J., and Zhang, D. (2013). MYB56 encoding a R2R3 MYB transcription factor regulates seed size in Arabidopsis thaliana. J Integr Plant Biol 55, 1166-1178. doi:10.1111/jipb.12094 |
| 11 | *GmMYB185* | 778044 | *GLYMA_19G257400* |  |
| 12 | *GmMYB92* | 100101857 | *GLYMA_16G023000* | Sun, Q., Lu, H., Zhang, Q., Wang, D., and Chen, J., et al. (2021). Transcriptome sequencing of wild soybean revealed gene expression dynamics under low nitrogen stress. J Appl Genet. doi:10.1007/s13353-021-00628-1 |
| 13 | *GmMYB20* | 100803624 | *GLYMA_11G215800* | Geng, P., Zhang, S., Liu, J., Zhao, C., and Wu, J., et al. (2020). MYB20, MYB42, MYB43, and MYB85 regulate phenylalanine and lignin biosynthesis during secondary cell wall formation. Plant Physiol 182, 1272-1283. doi:10.1104/pp.19.01070 |
| 14 | *GmMYB106* | 100792994 | *GLYMA_02G110200* | Liu, H., Fangfang, N., Youshun, L., Shuang, W., and Liyuan, C., et al. (2021). MYB106 is a negative regulator and a substrate for CRL3~(BPM) E3 ligase in regulating flowering time in Arabidopsis thaliana. Journal of Integrative Plant Biology 63, 1104-1119. |
| 15 | *GmMYB86* | 778182 | *GLYMA_03G227700* |  |
| 16 | *GmMYB64* | 100037467 | *GLYMA_19G222200* | Rabiger, D.S., and Drews, G.N. (2013). MYB64 and MYB119 are required for cellularization and differentiation during female gametogenesis in Arabidopsis thaliana. PLoS Genet 9, e1003783. doi:10.1371/journal.pgen.1003783 |
| 17 | *GmMYB4* | 100810385 | *GLYMA_12G199100* | Agarwal, P., Mitra, M., Banerjee, S., and Roy, S. (2020). MYB4 transcription factor, a member of R2R3-subfamily of MYB domain protein, regulates cadmium tolerance via enhanced protection against oxidative damage and increases expression of PCS1 and MT1C in Arabidopsis. Plant Sci 297, 110501. doi:10.1016/j.plantsci.2020.110501 |
| 18 | *GmMYB29B2* | 100807569 | *GLYMA_20G209700* |  |
| 19 | *GmMYB80* | 100800764 | *GLYMA_18G071600* | Phan, H.A., Iacuone, S., Li, S.F., and Parish, R.W. (2011). The MYB80 transcription factor is required for pollen development and the regulation of tapetal programmed cell death in Arabidopsis thaliana. Plant Cell 23, 2209-2224. doi:10.1105/tpc.110.082651 |
| 20 | *GmMYBJ2* | 100807020 | *GLYMA_04G170100* |  |
| 21 | *GmMYB60* | 778080 | *GLYMA_18G259100* | Oh, J.E., Kwon, Y., Kim, J.H., Noh, H., and Hong, S.W., et al. (2011). A dual role for MYB60 in stomatal regulation and root growth of Arabidopsis thaliana under drought stress. Plant Mol Biol 77, 91-103. doi:10.1007/s11103-011-9796-7 |
| 22 | *GmMYB53* | 780542 | *GLYMA_13G063200* |  |
| 23 | *GmMYB81* | 778175 | *GLYMA_14G086500* | Oh, S.A., Hoai, T., Park, H.J., Zhao, M., and Twell, D., et al. (2020). MYB81, a microspore-specific GAMYB transcription factor, promotes pollen mitosis I and cell lineage formation in Arabidopsis. Plant J 101, 590-603. doi:10.1111/tpj.14564 |
| 24 | *GmMYB17* | 100807142 | *GLYMA_10G236400* |  |
| 25 | *GmMYB149* | 778091 | *GLYMA_08G293300* |  |
| 26 | *GmMYB51* | 778077 | *GLYMA_03G078000* | Frerigmann, H., and Gigolashvili, T. (2014). MYB34, MYB51, and MYB122 distinctly regulate indolic glucosinolate biosynthesis in Arabidopsis thaliana. Mol Plant 7, 814-828. doi:10.1093/mp/ssu004 |
| 27 | *GmMYB109* | 778049 | *GLYMA_17G121000* | So, W.M., Huque, A., Shin, H.Y., Kim, S.Y., and Shin, J.S., et al. (2020). AtMYB109 negatively regulates stomatal closure under osmotic stress in Arabidopsis thaliana. J Plant Physiol 255, 153292. doi:10.1016/j.jplph.2020.153292 |
| 28 | *GmMYB50* | 778197 | *GLYMA_11G052100* |  |
| 29 | *GmMYB68* | 778037 | *GLYMA_04G042300* |  |
| 30 | *GmMYB70* | 778174 | *GLYMA_17G237900* | Cao, H., Chen, J., Yue, M., Xu, C., and Jian, W., et al. (2020). Tomato transcriptional repressor MYB70 directly regulates ethylene-dependent fruit ripening. Plant J 104, 1568-1581. doi:10.1111/tpj.15021 |
| 31 | *GmMYB107* | 778177 | *GLYMA_08G189900* | Gou, M., Hou, G., Yang, H., Zhang, X., and Cai, Y., et al. (2017). The MYB107 transcription factor positively regulates suberin biosynthesis. Plant Physiol 173, 1045-1058. doi:10.1104/pp.16.01614 |
| 32 | *GmMYB184* | 778045 | *GLYMA_08G042100* |  |
| 33 | *GmMYB76* | 778040 | *GLYMA_02G009800* | Duan, S., Jin, C., Li, D., Gao, C., and Qi, S., et al. (2017). MYB76 inhibits seed fatty acid accumulation in arabidopsis. Front Plant Sci 8, 226. doi:10.3389/fpls.2017.00226 |
| 34 | *GmMYB57* | 778034 | *GLYMA_14G191700* | Cheng, H., Song, S., Xiao, L., Soo, H.M., and Cheng, Z., et al. (2009). Gibberellin acts through jasmonate to control the expression of MYB21, MYB24, and MYB57 to promote stamen filament growth in Arabidopsis. PLoS Genet 5, e1000440. doi:10.1371/journal.pgen.1000440 |
| 35 | *GmMYB173* | 778058 | *GLYMA_11G211643* |  |
| 36 | *GmMYB133* | 778164 | *GLYMA_07G066100* | Bian, S., Li, R., Xia, S., Liu, Y., and Jin, D., et al. (2018). Soybean CCA1-like MYB transcription factor GmMYB133 modulates isoflavonoid biosynthesis. Biochem Biophys Res Commun 507, 324-329. doi:10.1016/j.bbrc.2018.11.033 |
| 37 | *GmMYB136* | 778054 | *GLYMA_09G131400* |  |
| 38 | *GmMYB183* | 778056 | *GLYMA_06G187600* |  |
| 39 | *GmMYB143* | 778057 | *GLYMA_04G177300* |  |
| 40 | *GmMYB175* | 780536 | *GLYMA_17G094400* |  |
| 41 | *GmMYB3* | 100791381 | *GLYMA_01G207600* | Tominaga-Wada, R., and Wada, T. (2016). The ectopic localization of CAPRICE LIKE MYB3 protein in Arabidopsis root epidermis. J Plant Physiol 199, 111-115. doi:10.1016/j.jplph.2016.05.014 |
| 42 | *GmMYB13* | 547459 | *GLYMA_20G184200* |  |
| 43 | *GmMYB98* | 778047 | *GLYMA_19G061600* | Kasahara, R.D., Portereiko, M.F., Sandaklie-Nikolova, L., Rabiger, D.S., and Drews, G.N. (2005). MYB98 is required for pollen tube guidance and synergid cell differentiation in Arabidopsis. Plant Cell 17, 2981-2992. doi:10.1105/tpc.105.034603 |
| 44 | *GmMYB182* | 778048 | *GLYMA_19G061300* | Yoshida, K., Ma, D., and Constabel, C.P. (2015). The MYB182 protein down-regulates proanthocyanidin and anthocyanin biosynthesis in poplar by repressing both structural and regulatory flavonoid genes. Plant Physiol 167, 693-710. doi:10.1104/pp.114.253674 |
| 45 | *GmMYB108* | 100805341 | *GLYMA_09G032100* | Mandaokar, A., and Browse, J. (2009). MYB108 acts together with MYB24 to regulate jasmonate-mediated stamen maturation in Arabidopsis. Plant Physiol 149, 851-862. doi:10.1104/pp.108.132597 |
| 46 | *GmMYB52* | 778032 | *GLYMA_15G019400* |  |
| 47 | *GmMYB127* | 778052 | *GLYMA_08G029400* |  |
| 48 | *GmMYB93* | 778163 | *GLYMA_05G062300* | Gibbs, D.J., and Coates, J.C. (2014). AtMYB93 is an endodermis-specific transcriptional regulator of lateral root development in arabidopsis. Plant Signal Behav 9, e970406. doi:10.4161/15592316.2014.970406 |
| 49 | *GmMYB158* | 780554 | *GLYMA_01G0386002* |  |
| 50 | *GmMYB138* | 778178 | *GLYMA_02G026300* |  |
| 51 | *GmMYB150* | 778092 | *GLYMA_06G201500* |  |
| 52 | *GmMYB12A* | 547458 | *GLYMA_03G221700* |  |
| 53 | *GmMYB159* | 100814923 | *GLYMA_07G216000* |  |
| 54 | *GmMYB178* | 778038 | *GLYMA_17G1671001* |  |
| 55 | *GmMYB121* | 778159 | *GLYMA_15G176000* |  |
| 56 | *GmMYB112* | 780556 | *GLYMA_01G190100* | Lotkowska, M.E., Tohge, T., Fernie, A.R., Xue, G.P., and Balazadeh, S., et al. (2015). The Arabidopsis Transcription Factor MYB112 Promotes Anthocyanin Formation during Salinity and under High Light Stress. Plant Physiol 169, 1862-1880. doi:10.1104/pp.15.0060 |
| 57 | *GmMYB45* | 100780267 | *GLYMA_12G066000* |  |
| 58 | *GmMYB139* | 778055 | *GLYMA_13G333200* |  |
| 59 | *GmMYB124* | 778051 | *GLYMA_06G036800* |  |
| 60 | *GmMYB82* | 778041 | *GLYMA_15G034500* | Liang, G., He, H., Li, Y., Ai, Q., and Yu, D. (2014). MYB82 functions in regulation of trichome development in Arabidopsis. J Exp Bot 65, 3215-3223. doi:10.1093/jxb/eru179 |
| 61 | *GmMYB177* | 778060 | *GLYMA_14G210600* |  |
| 62 | *GmMYB142* | 778061 | *GLYMA_19G127900* |  |
| 63 | *GmMYB164* | 100804049 | *GLYMA_14G074500* |  |
| 64 | *GmMYB83* | 778063 | *GLYMA_02G254200* | McCarthy, R.L., Zhong, R., and Ye, Z.H. (2009). MYB83 is a direct target of SND1 and acts redundantly with MYB46 in the regulation of secondary cell wall biosynthesis in Arabidopsis. Plant Cell Physiol 50, 1950-1964. doi:10.1093/pcp/pcp139 |
| 65 | *GmMYB114* | 778086 | *GLYMA_03G261800* | Ni, J., Bai, S., Zhao, Y., Qian, M., and Tao, R., et al. (2019). Ethylene response factors Pp4ERF24 and Pp12ERF96 regulate blue light-induced anthocyanin biosynthesis in 'Red Zaosu' pear fruits by interacting with MYB114. Plant Mol Biol 99, 67-78. doi:10.1007/s11103-018-0802-1 |

A comparison of *GmMYB* genes identified in current study with those of previous study.

**Table S5. *GsMYB7* downstream candidate gene information**

| **Serial Number** | **Gene Symbol** | **Transcript ID** | **Locus tag** | **Descriptions** | **[Related articles in PubMed](https://www.ncbi.nlm.nih.gov/gene/?term=Glyma.07g140000)** |
| --- | --- | --- | --- | --- | --- |
| 1 | LOC100818413 | XM_006592709.2 | *GLYMA_12G187600* | PREDICTED:Glycine max protein YLS9 (LOC100818413), mRNA |  |
| 2 | LOC100817436 | XM_014768463.1 | *GLYMA_16G164800* | PREDICTED: Glycine max ethylene-responsive transcription factor ABR1-like (LOC100817436), mRNA |  |
| 3 | LOC100786004 | XM_003525073.3 | *GLYMA_05G191400* | PREDICTED: Glycine max BTB/POZ domain-containing protein At2g13690-like (LOC100786004), mRNA |  |
| 4 | LOC547578 | NM_001251730.1 | *GLYMA_07G140000* | Glycine max glutathione S-transferase GST 7 (LOC547578), mRNA | McGonigle, B., Keeler, S.J., Lau, S.M., Koeppe, M.K., and O'Keefe, D.P. (2000). A genomics approach to the comprehensive analysis of the glutathione S-transferase gene family in soybean and maize. Plant Physiol 124, 1105-1120. doi:10.1104/pp.124.3.1105 |
| 5 | LOC100781111 | XM_003519095.3 | *GLYMA_02G202900* | PREDICTED: Glycine max CBL-interacting serine/threonine-protein kinase 6 (LOC100781111), mRNA |  |
| 6 | BZIP73A | NM_001248087.1 | *GLYMA_18G277100* | Glycine max bZIP transcription factor bZIP73A (BZIP73A), mRNA | Liao, Y., Zou, H.F., Wei, W., Hao, Y.J., and Tian, A.G., et al. (2008). Soybean GmbZIP44, GmbZIP62 and GmbZIP78 genes function as negative regulator of ABA signaling and confer salt and freezing tolerance in transgenic Arabidopsis. Planta 228, 225-240. doi:10.1007/s00425-008-0731-3 |
| 7 | LOC100803789 | XM_003536613.3 | *GLYMA_10G268900* | PREDICTED: Glycine max E3 ubiquitin-protein ligase RHA2A-like (LOC100803789), mRNA |  |
| 8 | AOS2 | NM_001249516.1 | *GLYMA_17G246500* | Glycine max allene oxide synthase (AOS2), mRNA | Wu, J., Wu, Q., Wu, Q., Gai, J., and Yu, D. (2008). Constitutive overexpression of AOS-like gene from soybean enhanced tolerance to insect attack in transgenic tobacco. Biotechnol Lett 30, 1693-1698. doi:10.1007/s10529-008-9742-1 |
| 9 | LOC100803787 | XM_014771166.1 | *GLYMA.18G212200* | PREDICTED: Glycine max CBL-interacting protein kinase 2-like (LOC100803787), transcript variant X1, mRNA |  |
