# Supplementary material for: GsMYB7 encoding a R2R3-type MYB transcription factor enhances the tolerance to aluminum stress in soybean (Glycine max L.)
Source: BMC Genomics. 2022 Jul 22;23:529. doi: 10.1186/s12864-022-08744-w (PMC9306046; doi:10.1186/s12864-022-08744-w)
Supplement: Supplementary file 3 — Additional file 3: Table S10. Bar primers were used to identify the transgenic lines by PCR. [file 12864_2022_8744_MOESM3_ESM.docx]

**Table S10. Bar primers were used to identify the transgenic lines by PCR**


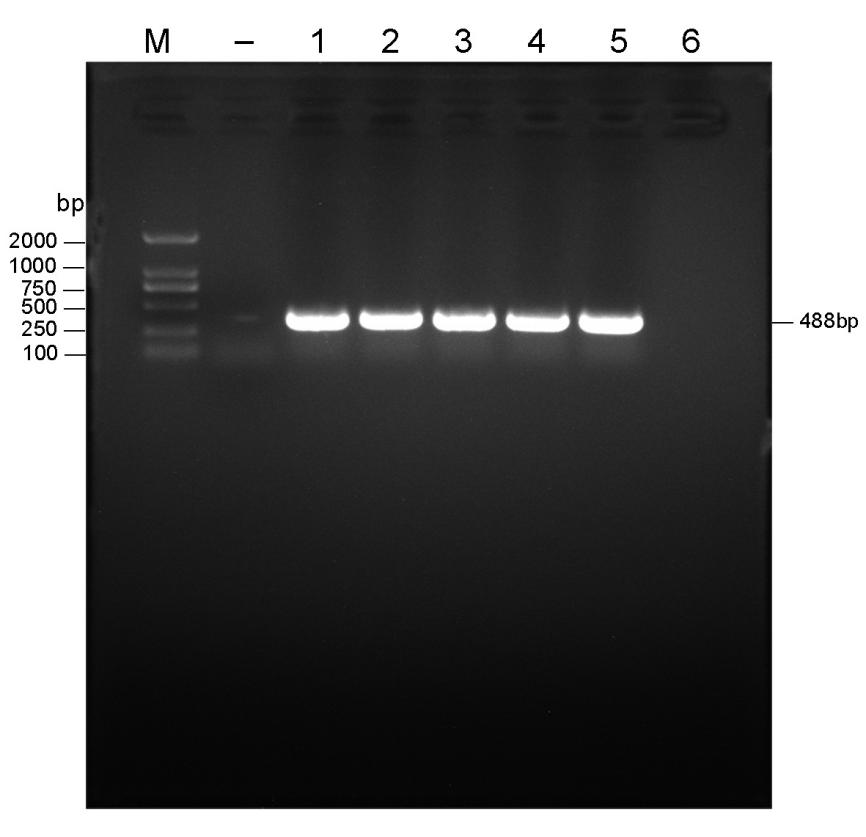


M：DL2000 marker； -: Wild type, HC6; 1, 2, 3, 4, 5: *GsMYB7* transgenic T_3_ soybean, corresponding to L1, L2, L3, L4, L5 respectively.
